# Rapid and precise identification of bloodstream infections using a pre-treatment protocol combined with high-throughput multiplex genetic detection system

**DOI:** 10.1186/s12879-022-07793-6

**Published:** 2022-11-08

**Authors:** Jinghao Zhang, Feng Yang, Zhaoyang Sun, Yi Fang, Haowei Zhu, Dijun Zhang, Xianping Zeng, Wenjian Liu, Tao Liu, Yixin Liu, Wenjing Chi, Su Wang, Li Ding, Yong Wu, Yanmei Zhang, Hu Zhao

**Affiliations:** 1 Department of Laboratory Medicine, Huadong Hospital, Fudan University, 221 West Yan’an Rd., Shanghai, 200040 China; 2 Shanghai Key Laboratory of Clinical Geriatric Medicine, Huadong Hospital, Fudan University, Shanghai, China; 3 Research Center on Aging and Medicine, Huadong Hospital, Fudan University, Shanghai, China; 4 Ningbo Health Gene Technologies Co., Ltd, Ningbo, Zhejiang China; 5 Department of Infectious Diseases, Sir Run Run Shaw Hospital, Zhejiang University, Hangzhou, Zhejiang China

**Keywords:** Bloodstream infection, Identification, High-throughput multiplex genetic detection system, Pre-treatment protocol, PCR inhibitor removal

## Abstract

**Background:**

Bloodstream infection (BSI) is a life-threatening condition with high morbidity and mortality rates worldwide. Early diagnosis of BSI is critical to avoid the unnecessary application of antimicrobial agents and for proper treatment. However, the current standard methods based on blood culture are time-consuming, thus failing to provide a timely etiological diagnosis of BSI, and common PCR-based detection might be inhibited by matrix components.

**Methods:**

The current study explored an integrated pre-analytical treatment protocol for whole blood samples, wherein pathogens are enriched and purified by incubation and concentration, and inhibitors are inactivated and removed. Further, this study developed and evaluated a novel high-throughput multiplex genetic detection system (HMGS) to detect 24 of the most clinically prevalent BSI pathogens in blood culture samples and pre-treated whole blood samples. The specificity and sensitivity were evaluated using related reference strains and quantified bacterial/fungal suspensions. The clinical utility of BSI-HMGS combined with the pre-analytical treatment protocol was verified using blood cultures and whole blood samples.

**Results:**

The combined pre-treatment protocol and BSI-HMGS was highly specific for target pathogens and possessed a low detection limit for clinical whole blood samples. The pre-treatment protocol could deplete the PCR inhibitors effectively. For blood culture samples, the current method showed 100.0% negative percent agreements and > 87.5% positive percent agreements compared to the reference results based on blood culture findings. For whole blood samples, the current method showed 100.0% negative percent agreements and > 80.0% positive percent agreements compared to the reference results for most pathogens. The turnaround time was ≤ 8 h, and all the procedures could be conducted in a general clinical laboratory.

**Conclusion:**

The BSI-HMGS combined with the pre-treatment protocol was a practical and promising method for early and precise detection of BSIs, especially for areas without access to advanced medical facilities.

**Supplementary Information:**

The online version contains supplementary material available at 10.1186/s12879-022-07793-6.

## Background

Bloodstream infection (BSI) is one of the most severe systemic infections and a leading cause of morbidity and mortality worldwide [1, 2]. Bacterial BSIs are associated with a mortality rate greater than 20%, and in *Candida* BSIs the mortality rate reaches 50% [3–5]. As reported previously, a delay in appropriate antimicrobial therapy may result in a mortality increase of 7.6% for each hour in the case of septic shock [6]. Thus, early and precise diagnosis of BSI is essential for improving the clinical outcome of BSIs.

Blood culture is still the “gold standard” for BSI diagnosis and can identify various pathogens in blood [7]. However, several limitations influence the diagnostic efficiency of blood culture even when a bloodstream infection is strongly suspected. First, blood culture is time-consuming and takes at least 24–48 h to isolate and identify bacteria or *Candida* in positive samples and requires five days for culture-negative samples [8]. Secondly, the pathogen loads are low, and some slow-growing or fastidious pathogens are difficult for incubation and subsequent identification [9]. Third, blood cultures are frequently contaminated with increasing culture duration, which may result in unnecessary antimicrobial therapy, prolonged length of stay, and increased healthcare costs [10]. Thus, other rapid and precise approaches are urgently needed for BSI diagnosis.

Molecular methods based on polymerase chain reaction (PCR) technology have been applied for the direct and rapid detection of causative agents in infectious diseases [11–13]. The development of multiplex genetic detection systems has enabled the simultaneous detection of different pathogens in a single panel, which have been successfully applied for diagnosing infectious diseases including diarrhoea, *Helicobacter pylori* infection, and respiratory infections [14, 15]. Consequently, there is increasing interest in using molecular methods directly for analysing whole blood, which could break through the bottleneck of culture and shorten the turnaround time. However, typically, organisms circulating in the blood of patients with BSI can be as low as 10 colony forming units per millilitre (CFU/mL) [16, 17], and these concentrations of pathogens in whole blood cannot satisfy the requirements of ordinary PCR-based detection [18]. Moreover, another common limitation of PCR-based methods that results in failed amplification is the presence of PCR inhibitors in blood, such as non-target DNA [19], immunoglobulins [20], and other possible inhibitors in the sample matrix components of blood culture bottles. Even for the positive blood culture samples, the inhibitors may need to be diluted, inactivated, or removed before successful identification. Therefore, pre-analytical protocols are required for pathogen enrichment and inhibitor disposal.

In this study, we examined a pre-analytical treatment protocol for whole blood samples involving incubation for enriching pathogenic microorganisms, concentration and purification for pathogens in blood culture bottles, and inactivation and removal of inhibitors for subsequent PCR-based tests. We also developed and evaluated a high-throughput multiplex genetic detection system (BSI-HMGS) for simultaneously detecting 24 of the clinically most prevalent BSI pathogens from blood culture samples and pre-treated whole blood samples. We aimed to develop a rapid and convenient protocol for the molecular detection of pathogens from suspected BSIs samples in a general clinical laboratory.

## Methods

### Samples

(i) *Clinical samples* Analytical clinical samples included conventional blood culture samples and blood samples that had been treated using a pre-analytical treatment protocol (detailed under “Pre-treatment protocol” in the Materials and methods section), and both of these were collected according to the Chinese guidelines of Specimen collection and transport in clinical microbiology (WS/T 640-2018). Versa TREK Redox bottles and the Versa TREK automated blood culture system (Thermo Fisher Scientific, MA, USA) were used for blood culture. Blood culture samples were collected consecutively from January 2019 to December 2020 at Huadong hospital based on the routine identification results. Inclusion criteria were as follows: (1) all patients had a positive blood culture for at least one of the target pathogens, (2) clinical manifestations of bloodstream infection, and (3) inpatients with complete clinical microbiological data for analysis. Repeat blood cultures within 14 days of the initial blood culture and yielding an identical pathogen were excluded. In total, 426 clinical positive blood culture samples were collected in the current study. Moreover, 100 clinical negative blood culture samples were also used to test the performance of the new methods. Meanwhile, duplicate sets of blood samples were collected from 214 enrolled patients who volunteered to provide double samples. One set of these samples was subjected to conventional testing and the other was subjected to the pre-analytical treatment protocol and identification using the BSI-HMGS.

(ii) *Simulated samples* Positive whole blood samples were simulated to evaluate the efficiency of the pre-analytical treatment protocol and BSI-HMGS. The simulation workflow was as follows: (1) the bacterial/fungal reference strains were incubated on Columbia blood agar plates (*Haemophilus influenzae* on chocolate agar plate) and cultured at 35 °C in an atmosphere of 5% CO_2_ for 24 h. Then the colonies were picked from the solid mediums and diluted with 0.45% NaCl to reach turbidity of 0.5 McFarland turbidity unit (MCF) using a DensiCHEK turbidimeter (BioMerieux, Lyon, France). These suspensions were serially diluted to the desired concentrations and incubated on appropriate solid culture mediums until colony-forming units (CFU) were visible (the bacterial suspension was totally diluted about 1.0 × 10^5^ fold and the fungal suspension was totally diluted about 1.0 × 10^3^ fold). The final countable colonies were shown in Additional file 1: Fig. S1. Accurate CFU/mL of each 0.5 MCF suspension was determined by plate count. (2) Then diluted each 0.5 MCF suspension with 0.45% NaCl and spiked in sterile defibrinated sheep blood (Yuanye Bio-Technology, Shanghai, China) to yield a load 10 CFU/mL of simulated whole blood sample [21]. (3) Finally, injected 10 mL of the spiked blood samples to Versa TREK Redox bottles and loaded the bottles in the Versa TREK blood culture system for further pre-analytical treatment. The concentration–response curves, including the information of time to positivity, from the Versa TREK system for these bottles were shown as Additional file 1: Fig. S2, which was consistent with that reported in previous studies [22]. Five clinical isolates from different patients were used as simulation bacteria/fungi for each pathogen. In total, 720 simulated samples (30 for each organism) were obtained in this study.

(iii) *Reference strains* Twenty-four reference strains relative to the pathogens in the panel were used to establish and optimize the BSI-HMGS, including *Klebsiella pneumoniae* (ATCC700603), *Burkholderia cepacia* (ATCC25416), *Proteus mirabilis* (ATCC12453), *Moraxella catarrhalis* (ATCC25238), *Serratia marcescens* (ATCC8100), *H. influenzae* (ATCC49766), *Pseudomonas aeruginosa* (ATCC27853), *Enterobacter cloacae* (ATCC13047), *Escherichia coli* (ATCC25922), *Acinetobacter baumannii* (ATCC19606), *Stenotrophomonas maltophilia* (ATCC17666), *Salmonella typhimurium* (ATCC14028), *Staphylococcus hominis* (ATCC27844), *Staphylococcus aureus* (ATCC29213), *Streptococcus salivarius* (ATCC13419), *Streptococcus pneumoniae* (ATCC49619), *Streptococcus pyogenes* (ATCC12344), *Streptococcus agalactiae* (ATCC12388), *Enterococcus faecalis* (ATCC29212), *Enterococcus faecium* (ATCC35667), *Candida albicans* (ATCC14053), *Candida tropicalis* (ATCC66029), *Candida parapsilosis* (ATCC22019) and *Candida glabrata* (ATCC15126).

### Conventional tests

Clinical blood samples were collected in the Versa TREK Redox bottles and incubated on the Versa TREK system (Thermo Fisher Scientific). Positive clinical blood cultures were transferred to Columbia blood agar, Chocolate agar, and Sabouraud’s agar. Negative samples were incubated for at least 5 days in the automated blood culture system. All incubation and sub-cultivation procedures were performed following the Clinical and Laboratory Standards Institute (CLSI, M47-A) guidelines. The colonies on agar plates were identified by Matrix-Assisted Laser Desorption Ionization time-of-flight mass spectrometry (MALDI-TOF MS, BioMerieux) after 4, 6, 24 and 48 h, and these results were used for comparison.

### Application of BSI-HMGS

BSI-HMGS was performed from positive blood cultures asap they were indicated positive by the Versa Trek System or from the selected negative cultures; BSI-HMGS was performed from whole blood samples after a pre-treatment protocol. If the BSI-HMGS result was not consistent with the corresponding result in MALDI-TOF MS, sequencing (Shenggong Bioengineering Technology, Shanghai, China) was performed for verification and this result was determined as the final reference result. If BSI-HMGS yielded the same detection result as MALDI-TOF MS, this result was directly determined as the reference result.

### Pre-treatment protocol

To accelerate BSI pathogen identification using PCR-based methods, a protocol for the enrichment of pathogens from whole blood sample and depleting PCR inhibitors prior to extraction of microbial DNA was developed. The details are as follows:

(i) *Incubation* To increase pathogen load in whole blood samples from patients with suspected BSI, one set of the blood samples from the enrolled patients was collected in Versa TREK Redox bottles and incubated on the Versa TREK automated blood culture system for 5 h, as were the simulated whole blood samples.

(ii) *Concentration* The amount of blood sample utilized for nucleic acid extraction can affect the detection sensitivity of PCR-based methods. Therefore, we concentrated the pathogen from 5 mL samples for the subsequent BSI-HMGS assay. First, 5 mL of suspension from step (i) was added to a Serum separation tube (SST) (Becton, Dickinson and Company, NJ, USA). Second, the SST was centrifuged at 900*g* for 10 min, and the supernatant was discarded; the pathogen cells would be concentrated on the surface of the separation gel. Third, 1 mL of 0.45% NaCl was added to the SST, and shaken intermittently using a vortex mixer for 15 s to resuspend the pathogen cells. Finally, the suspension above the separation gel was transferred to a 2 mL centrifugation tube. The concentration was performed for the whole blood samples after incubation and for all the blood cultures tested.

(iii) *Inhibitor removal* The existence of PCR inhibitors in blood culture bottles, including 3 dominant vendors Versa TREK Redox, BACTEC Vials, and BacT/AlerT, has been proven in the following studies (*detailed under “Confirmation of the presence of PCR inhibitors in the blood culture system” in the Materials and methods section*), even after nucleic acid extraction. Thus, the 1 mL suspension obtained in step (ii) was purified to remove any inhibitors. First, 1 mL of selective lysis buffer (Fosun Pharmaceutical, Shanghai, China), mainly containing 10% sodium dodecyl sulphate (SDS) and 1 μL glycogen (Yuanye Bio-Technology, 20 mg/mL) as a carrier for the precipitation of DNA, was added to the suspension, agitated for 15 s, and centrifuged at 15,850*g* for 1 min. Then, the supernatant was discarded and the sediment was washed using 1 mL wash buffer (Fosun Pharmaceutical), mainly containing Tris buffer (PH 8.0), in a manner similar to that used for the lysis buffer. The inhibitor removal was also performed for the whole blood samples after incubation and for all the blood cultures tested. The effect of the 10% SDS treatment on the target organisms was further evaluated to confirm its availability: each organism suspension was treated with the lysis buffer and 0.45% NaCl in the same way above, respectively, then the sediment was examined by smear microscopy.

### Nucleic acid extraction

Total nucleic acids were extracted from a 300 μL suspension of blood cultures and the pre-treated sample indicated above, and the cells were broken by vortexing for 3 min with glass beads (100 mg, 0.25 mm in diameter). Then 10 μL proteinase K (1 mg/mL) was added to each sample, and the Nucleic Acid Extraction or Purification Kit (HEALTH Gene Technologies, Ningbo, China) was used according to the manufacturer’s instructions. This kit was based on the principle of guanidine isolation and silica dioxide adsorption using a magnetic bead-based method. Meanwhile, a modified fragment of the kanamycin resistance gene (*Kanr*) was inserted into the pcDNA3.1 vector to generate a fusion plasmid that served as internal control (IC) for the detection system, and the IC plasmid (3 μL, 1.0 × 10^5^ copies/μL) was added to every clinical sample prior to extraction as a positive control throughout the whole experiment. The extracts were eluted with 60 μL of DNase/Rnase-free H_2_O (ddH_2_O). The concentration of each extract was determined using a Thermo Nanodrop 2000 spectrophotometer (Thermo Fisher Scientific). The extracts were stored at − 80 °C until further analysis. Another nucleic acid extraction kit (Tiangen Biotech, Beijing, China) based on an absorbent column principle was also tested to verify its DNA purification efficiency.

### BSI-HMGS primer design

Twenty-five pairs of primers targeting one IC gene and twenty-four conserved genes of specific pathogens, including 12 g-negative bacterial species/complex (*K. pneumoniae*, *B. cepacia*, *P. mirabilis*, *M. catarrhalis*, *S. marcescens*, *H. influenzae*, *P. aeruginosa*, *Enterobacter cloacae* complex, *E. coli*, *A. baumannii*, *S. maltophilia* and *S. enterica*), 8 g-positive bacterial genus/species (*Staphylococcus*, *S. aureus*, *Streptococcus*, *S. pneumoniae*, *S. pyogenes, S. agalactiae, E. faecalis*, and *E. faecium*), and 4 fungi (*C. albicans*, *C. tropicalis*, *C. parapsilosis,* and *C. glabrata*), were designed using Premier 5.0 software (Premier Biosoft International, CA, USA) (Table 1). To achieve sufficient coverage of pathogens and to ensure that the primer pairs work appropriately, two pairs of primers were targeted at genus level and one was targeted at the bacterial complex, including primer pairs for *Staphylococcus* and *Streptococcus* genera and the *Enterobacter cloacae* complex, respectively. The primer pair for *Staphylococcus* targeted a specific gene present in *S. aureus*, *S. lugdunensis* and eight kinds of predominant coagulase-negative *staphylococci* including *S. capitis*, *S. epidermidis*, *S. hominis*, *S. haemolyticus*, *S. intermedius*, *S. saprophyticus*, *S. simulans*, and *S. warneri*. Thus, identification of *S. aureus* by BSI-HMGS would show two amplified peaks, one at the position for *S. aureus* and the other at the position for *Staphylococcus*. The primer pair for *Streptococcus* targeted a specific gene present in eight *Streptococcus* species including *S. agalactiae*, *S. pneumoniae*, *S. pyogenes*, *S. milleri*, *S. mitis*, *S. mutans*, *S. salivarius*, and *S. sanguinis*. Thus, identification of *S. agalactiae*, *S. pneumoniae*, and *S. pyogenes* by BSI-HMGS would also show two amplified peaks each, one at the position for *Streptococcus* and the other at their specific position. In addition, the *E. cloacae* complex in the BSI-HMGS panel includes seven species: *E. arbornii*, *E. cloacae*, *E. hokalii*, *E. kodoi*, *E. lukalii*, *E. mori*, and *E. nimipressuralis*, which would show a single peak at the position for *E. cloacae* complex when detected by BSI-HMGS. Overall, 42 bacteria and fungi could be detected with these 25 pairs of primers. All amplification product sizes ranged from 100 to 400 bp, with at least 3 base differences between each product. The peaks of primers dimers would show between 0 and 100 nt. The detailed primer information is listed in Table 1.

**Table 1:** Primer information used in BSI-HMGS

Target pathogen	Primer information	Concentration (μM/L)	Size (bp)
	Sequence		
Gram-negative bacteria			
*K. pneumoniae*	F: 5′-TGAGACTGTTCTGATTCCTGAT-3′	2	105
	R: 5′-GTCGATTCGTTTCACCGGA-3′	2	
*B. cepacia*	F: 5′-TGTATCGGGCTTACCATGCGAT-3′	1	123
	R: 5′-GTCTATACTCTGCACTGACTTCCT-3′	1	
*P. mirabilis*	F: 5′-TGCGTCGACCAATCGTTGTTAT-3′	1	140
	R: 5′-GTAGTAGCTTTGAGACAGAAGGTGCTA-3′	1	
*M. catarrhalis*	F: 5′-TGACTGACTGCCACTGGTATGGATA-3′	1	150
	R: 5′-GTATTTGGCACCACGCATAAT-3′	1	
*S. marcescens*	F: 5′-TGACCGGTAACACTGATCG-3′	1	170
	R: 5′-GTGGTTTGCAGGTAAGGATC-3′	1	
*H. influenzae*	F: 5′-TGTCGTAACGCGTACTGTACCTG-3′	2	179
	R: 5′-GTGATTGAGTTCTCGTTGCATCTT-3′	2	
*P. aeruginosa*	F: 5′-TGCAACCGGACCTGTGGCGC-3′	1	188
	R: 5′-GCCTGGTAGTCTTCGGCACT-3′	1	
*E. cloacae *complex	F: 5′-TGCACGATGATGAAACCAAATGCGAC-3′	1	203
	R: 5′-GTAGACCATTGTAGCATTCTGTTTCCGGT-3	1	
*E. coli*	F: 5′-TGCCTGGACGGATGAAAGTAGACA-3′	1	213
	R: 5′-GTTCAGGAAGGAGCCGATATCATCTCTGA-3′	1	
*A. baumannii*	F: 5′-TGACTCTTTAATCCGCCGTGAA-3′	1	267
	R: 5′-GTGCATCGTGGCTAATTTGTG-3′	1	
*S. maltophilia*	F: 5′-TGATAGGAACAGAAGGTCGAGATCAA-3′	2	277
	R: 5′-GTCATTCGATCTGTGCCTTGTCGT-3′	2	
*S. enteritidis*	F: 5′-TGACGCGTCGTATTCGTTTACCAAAGC-3′	1	282
	R: 5′-GTATTCATGCTTGTAGGCAATATCGG-3′	1	
Gram-positive bacteria			
*Staphylococcus*	F: 5′-TCTCGTCAAATCGAATCWGC-3′	1	292
	R: 5′-GTTCACCACCCAATTCYTCACGTT-3′	1	
*S. aureus*	F: 5′-TGTATTCGCAGGTCCTTCA-3′	2	302
	R: 5′-GTTGACGAAACTGCGAGTGATTAAG-3′	2	
*Streptococcus*	F: 5′-TGACGTATTCCGTTCTAATACAGG-3′	1	220
	R: 5′-GTACCGTCCGTGATAKAGATATCCA-3′	1	
*S. pyogenes*	F: 5′-TGGAAGGCGAGTTAATCAGGTAG-3′	1.5	248
	R: 5′-GTAGAAGCCTGAGGAATCGCTA-3′	1.5	
*S. agalactiae*	F: 5′-TGCTGGGTTAGGAGTAGGTTTGTCAGC-3′	2	259
	R: 5′-GTTGATTGCGTGTACCTTGCGATAATG-3′	2	
*S. pneumoniae*	F: 5′-TGCTATACAATGGACGACCCTTTC-3′	2	345
	R: 5′-GTAGTTTGTCTGCTTCGACCTTTAT-3′	2	
*E. faecalis*	F: 5′-TGTCAAGACCAGTGTTCACGAA-3′	2	117
	R: 5′-GTGTCATGAAACGATGTTTGG-3′	2	
*E. faecium*	F: 5′-TGTAATCAGGAGTCGTTCTTGCGAT-3′	1.5	353
	R: 5′-GTGATATGGAAGTTTGTGCCGGTCATAT-3′	1.5	
Fungus			
*C. albicans*	F: 5′-TGTTGTTTGCCTTATTGGTTGCCT-3′	2	128
	R: 5′-GTCTTTGGATAACCGTTGATGGTAC-3′	2	
*C. tropicalis*	F: 5′-TGACTCTAGAAAGTCGCGTATTTC-3′	1	188
	R: 5′-GTCTGTGATTGAGAATGATCGCT-3′	1	
*C. parapsilosis*	F: 5′-TGCTGTTTGGGCGTCGTTG-3′	2	231
	R: 5′-GTCAAGGATAAGACGCGTATCTCCCTTC-3′	2	
*C. glabrata*	F: 5′-TGGTTGCACGATATACAGGGACAC-3′	1	332
	R: 5′-GTGCACACGCTGTATATGTTCTTGTT-3′	1	
IC			
IC	F: 5′-TGAACGTCTTACACCTCCTAAACA-3′	2	367
	R: 5′--GTCACGTTCCGGCATTGTCTTAT-3′-	2	

IC: internal control; F: forward; R: reverse

### Multiplex PCR and fragment analysis for the BSI-HMGS assay

Multiplex PCR was performed in a 10 μL volume containing 2 μL PCR Buffer (HEALTH Gene Technologies), 0.5 μL uracil-N-glycosylase (UNG), 0.4 μL Phoenix™ Hot Start Taq DNA Polymerase (Qiagen, Dusseldorf, Germany), 1 μL primer pool, 1.1 μL ddH_2_O, and 5 μL template. The primer pool comprised 25 mixed pairs of primers at different proportions to achieve optimum sensitivity for all targets (Table 1). The mixture was subjected to the following amplification conditions: 42 ℃ for 5 min; 94 ℃ for 8 min; 35 cycles of 94 ℃ for 30 s, 60 ℃ for 30 s, and 70 ℃ for 1 min; and 70 ℃ for 1 min. The products were stored at 4 °C until further use. The ddH_2_O was simultaneously detected as a negative control throughout the whole study. The multiplex PCR takes approximately 1 h and 24 min for 1–96 samples in one run.

The PCR products were then prepared for fragment analysis using the Genetic Analyzer 3500xL (Applied Biosystems, CA, USA). For each reaction, 1 μL PCR product was added to 9 μL highly deionized formamide containing 4% GeneScan 500 LIZ (Applied Biosystems) as an internal standard size marker, and analysed using high-resolution capillary gel electrophoresis. The data obtained was further analysed using the Gene Mapper ID*-X* software v1.6 (Applied Biosystems). Finally, the detection was considered positive when the peak height was greater than 500 relative fluorescence units (rfu). The fragment analysis takes approximately 40 min for 1–24 samples in one run.

### Plasmids construction

Plasmids of all the target genes were used to establish and optimize the BSI-HMGS. The process for construction and transformation of the 25 plasmids was as follows: (1) Ligation: 2 μL PCR products, 0.5 μL PMD-18T vector (Tiangen Biotech) and 2.5 μL solution I were mixed and kept under 16 ℃ for at least 45 min. (2) Transformation: 5 μL above product mixed with 1 mL competent cells (Tiangen Biotech) were processed as follows: incubation on ice-bath for 30 min; 42 ℃ thermal shock for 90 s; ice-bath for 2 min; 600 μL LB broth without ampicillin resistance was added and then placed in shaker under 37 ℃ for 45 min. (3) Inoculation: 200 μL transformed cells were inoculated under 37 ℃ for 12 h. (4) Identification: the target clone was identified by sequencing. DNA copy number was calculated by the following formula: [(6.02 × 10^23^ copy number/mol) × plasmid concentration (g/mL)/L × (MW g/mol) = copies/mL (MW: average molecular weight). Purification of plasmids was performed according to the manufacturer’s protocol.

### Confirmation of PCR inhibitors in the blood culture system

To determine whether the blood culture matrix components in the blood culture bottles contained PCR inhibitors, PCR-based detection was conducted to test the mixtures of the matrix components and specific bacterial suspensions. Six kinds of matrix components from three predominant vendors (aerobic/anaerobic) including Versa TREK Redox (Thermo Fisher Scientific), BACTEC Vials (Becton, Dickinson and Company, NJ, USA), and BacT/AlerT (BioMerieux), were tested. The matrix component in each bottle (100 μL) was mixed with specific bacterial suspension (200 μL, 1.0 × 10^4^ CFU/mL). Then the mixtures were extracted, amplified, and identified using common multiplex PCR Buffer and Taq DNA Polymerase (Roche, Basel, Switzerland), respectively. The mixture of bacterial suspension and matrix components from Versa TREK Redox, which was used for the blood culture of clinical samples in the current study, was subjected to the pre-analytical inhibitor removal protocol and then identified by the BSI-HMGS to verify its ability to counter possible inhibition. The mixture of bacterial suspension and ddH_2_O was also detected as a positive control. Two bacteria, *P. mirabilis* and *S. aureus*, were used for this validation. Three replicates were conducted for each test.

### Performance evaluation of BSI-HMGS and the pre-treatment protocol

The 24 reference strains in the panel as well as five pathogens detected in BSIs but not included in the BSI-HMGS panel, were tested to verify that the BSI-HMGS is specific to target pathogens. Quantified bacterial/fungal suspensions were tested to evaluate the limit of detection (LOD) of BSI-HMGS. Then, serial tenfold dilutions of plasmids mixed with equal amounts of templates were used to test the ability of BSI-HMGS to detect all pathogens simultaneously. Different two-plasmid mixtures (including gram-negative bacterial mixed with gram-positive bacterial plasmids, gram-negative bacterial with gram-negative bacterial plasmids, gram-positive bacterial with gram-positive bacterial plasmids, gram-negative bacterial with fungal plasmids, and gram-positive bacterial with fungal plasmids) were examined using BSI-HMGS to verify its capability of detecting polymicrobial infection. The concentrations of the two plasmids in each mixture had a tenfold difference. The performance of BSI-HMGS on blood culture samples was evaluated by comparison with the reference results. Clinical whole blood samples from the enrolled patients and simulated whole blood samples were used to test the performance of BSI-HMGS combined with the pre-treatment protocol.

### Statistics

The level of agreement between the assays was determined by calculating the overall percent agreement (OPA), positive percent agreement (PPA) and negative percent agreement (NPA) using the following formulas: OPA = (TP + TN)/(TP + FP + TN + FN); PPA = TP/(TP + FN); NPA = TN/(TN + FP) (where TP: true positive; FN: false negative; TN: true negative; FP: false positive, and all these true or false were determined based on the reference results). All statistical analyses were performed using Stata/SE 14.0 (Stata Corp College Station, TX, USA) for Mac.

## Results

### PCR inhibitors are present in various blood culture bottles

**Fig. 1:**
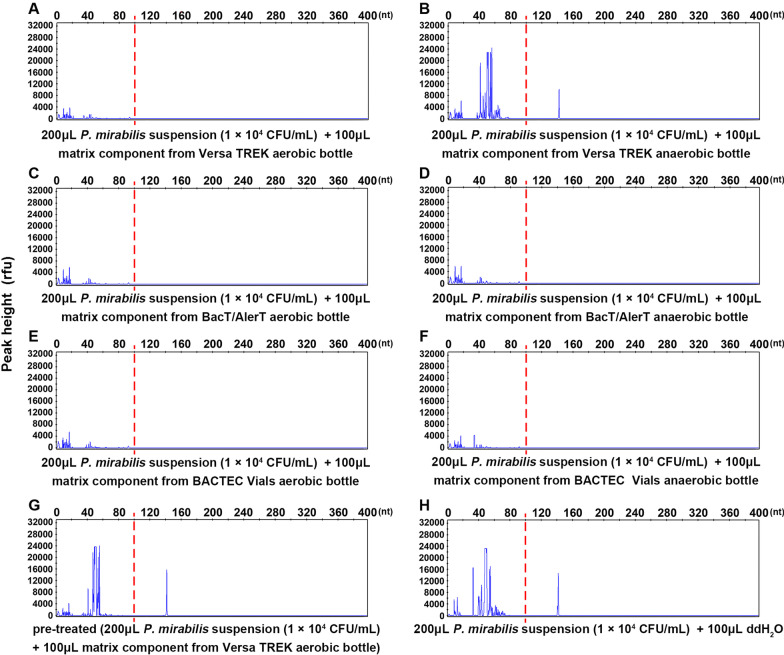
PCR inhibitors are present in various blood culture bottles. To explore the presence of PCR inhibitors in the matrix components of predominantly used blood culture bottles, mixtures of *P. mirabilis* suspension with matrix components from each bottle were extracted, amplified, and detected (**A**–**F**). To verify the counter inhibition efficacy of the pre-treatment protocol, the pre-treated mixture of *P. mirabilis* suspension and matrix components from Versa TREK Redox was examined (**G**). The mixture of *P. mirabilis* suspension and ddH_2_O was also examined as a positive control (**H**). The mixtures containing matrix components from most blood culture bottles showed no amplified peaks. Notably, no distinct non-specific primer dimer peaks were observed from 0 to 100 nt in these tests, which should have appeared in every successful amplification. In contrast, the pre-treated mixture and control mixture showed specific *p. mirabilis* peaks along with normal primer dimer peaks. Likewise, the mixture containing matrix component from the Versa TREK Redox anaerobic bottle showed correct peaks, which may result from its unique composition compared with other bottles. The dashed red lines denote the demarcation between primer dimer peaks (0–100 nt) and peaks of target pathogens (100–400 nt)

To determine whether PCR inhibitors were present in the matrix components of blood culture bottles, the matrix in six predominant commercial bottles were tested. The results showed no positive peaks when detecting the mixtures of bacterial suspension and matrix components from five bottles, including Versa TREK Redox aerobic bottles, BACTEC Vials aerobic and anaerobic bottles, and BacT/AlerT aerobic and anaerobic bottles (Fig. 1A, C–F). Notably, no distinct non-specific primer dimer peaks were observed around 0–100 nt, which would have appeared in every test with successful amplification. In contrast, specific peaks were observed in the reaction for the mixture of bacterial suspension and the matrix components from the Versa TREK Redox anaerobic bottle, the mixture that had been treated with the pre-analytical protocol, and the mixture of bacterial suspension and ddH_2_O (Fig. 1B, G, H), and regular primer dimer peaks were also observed along with the positive peaks, indicating the pre-treatment protocol could deplete the PCR inhibitors. Consistent results were obtained across different bacteria and replicates. Different nucleic acid extraction kits tested did not exhibit the ability to eliminate inhibition, and the extractions from some non-pre-treated positive blood culture samples needed to be diluted appropriately to achieve optimal results (data not shown).

Additionally, to evaluate the effect of 10% SDS treatment on different target organisms, each organism suspension was treated with the lysis buffer and 0.45% NaCl, respectively. The sediment was examined by smear microscopy. The results showed that compared with the control cells, some organisms treated with lysis buffer have slight morphological alterations, which mainly present as increased aggregation, and no apparent other morphological alteration was observed (Additional file 1: Fig. S3).

### BSI-HMGS is highly specific and sensitive for detecting BSI pathogens and allows simultaneous detection of 24 pathogens in a single reaction

**Fig. 2:**
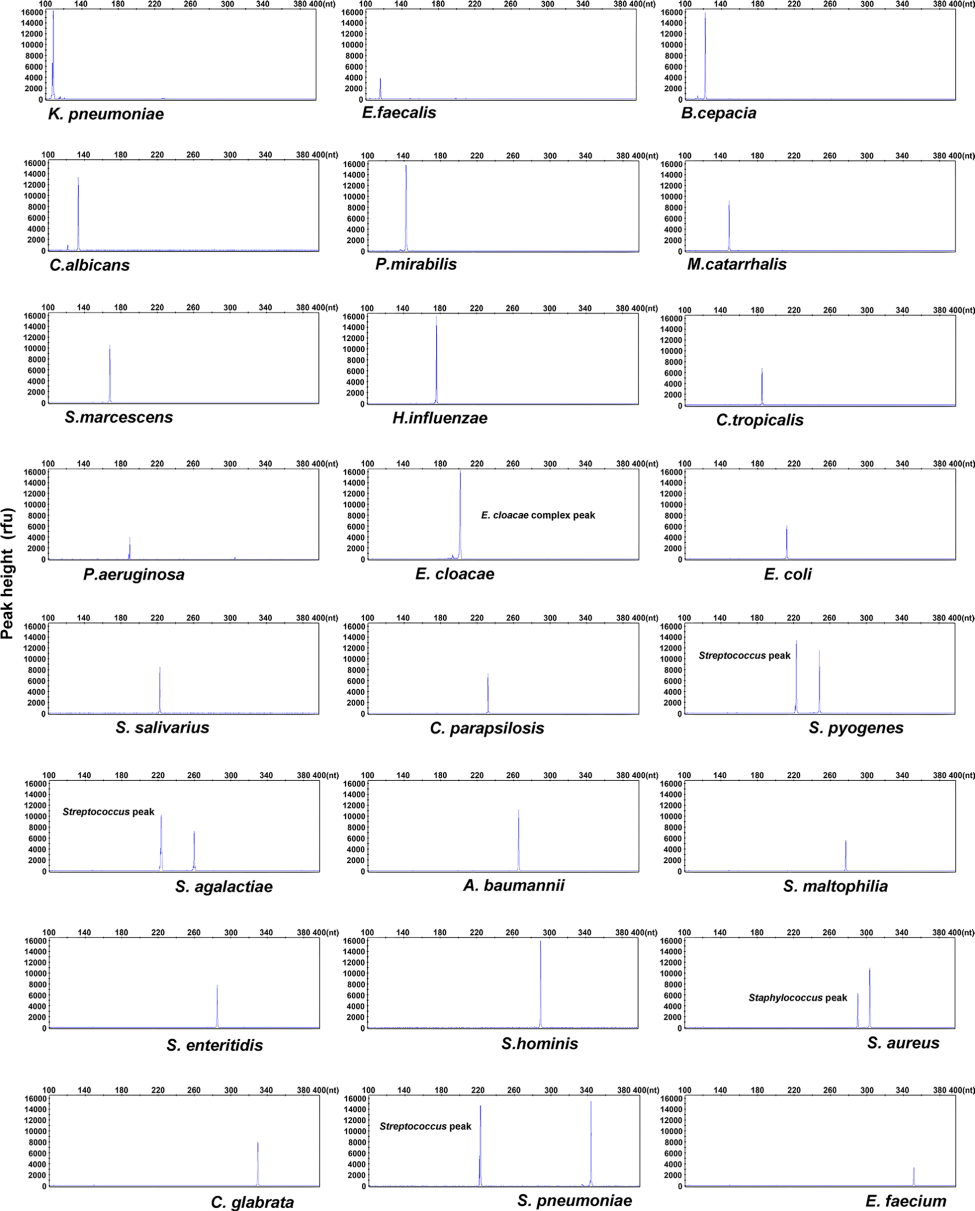
The BSI-HMGS can detect 24 target pathogens specifically. To evaluate the specificity of BSI-HMGS for the target pathogens, 24 reference strains in the panel were detected. Every target gene was specifically amplified without any non-specific amplification. The detection of *S. aureus*, *S. agalactiae*, *S. pneumoniae*, and *S. pyogenes* showed two peaks, among which one was specific for their species and the other corresponded to their genus. HMGS: high-throughput multiplex genetic detection system

**Fig. 3:**
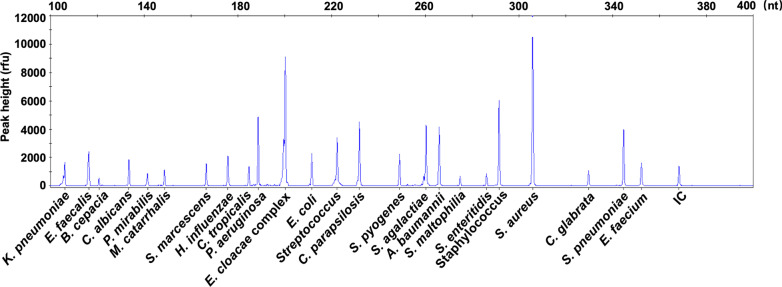
BSI-HMGS can simultaneously detect 24 pathogens in a single reaction. A mixture of all 25 plasmids, each at a concentration of 100 copies/μL, was detected by BSI-HMGS, and all specific peaks were found to be above the positive cut-off fluorescence signal of 500 rfu. No non-specific peak was observed, indicating that the optimized BSI-HMGS assay has a high level of sensitivity for simultaneously detecting the 24 target pathogen genes and the internal control gene in a single reaction. HMGS: high-throughput multiplex genetic detection system; IC: internal control

The specificity for each pathogen in the BSI-HMGS panel was evaluated using 24 corresponding reference strains. Specific peaks were obtained for the 24 pathogens (Fig. 2). Five pathogens that have been detected in BSIs but not included in BSI-HMGS, including *Listeria monocytogenes*, *Aeromonas hydrophila*, *Klebsiella oxytoca*, *Enterococcus avium* and *Candida guilliermondii*, and ddH_2_O were tested to rule out any non-specific amplification. No amplification except for IC was observed among these tests (see Additional file 1: Fig. S4). Next, to determine the LODs of BSI-HMGS in terms of quantified bacterial/fungal suspensions, 24 reference strains with tenfold serial dilutions, including 1.0 × 10^2^–1.0 × 10^5^ CFU/mL, were tested. All 24 targets could be detected at a concentration of 1.0 × 10^3^ CFU/mL, which showed a peak-height over the positive cut-off fluorescence signal of 500 rfu (see Additional file 1: Fig. S5), and some target pathogens could be detected at 1.0 × 10^2^ CFU/mL. Thus, the LOD of BSI-HMGS for bacterial/fungal suspensions was set at 1.0 × 10^3^ CFU/mL. These results demonstrated that the BSI-HMGS was highly specific and sensitive for detecting these 24 BSI pathogens. To evaluate the capability of detecting 25 targets, including the IC, in a single reaction using BSI-HMGS, a mixture of 25 corresponding plasmids was used. As shown in Fig. 3, the result was reliable when detecting a mixture of all 25 plasmids, each at a concentration of 100 copies/μL, and all specific peaks were above the positive cut-off fluorescence signal. No non-specific peak was observed.

### BSI-HMGS can robustly detect individual pathogens in polymicrobial mixtures

**Fig. 4:**
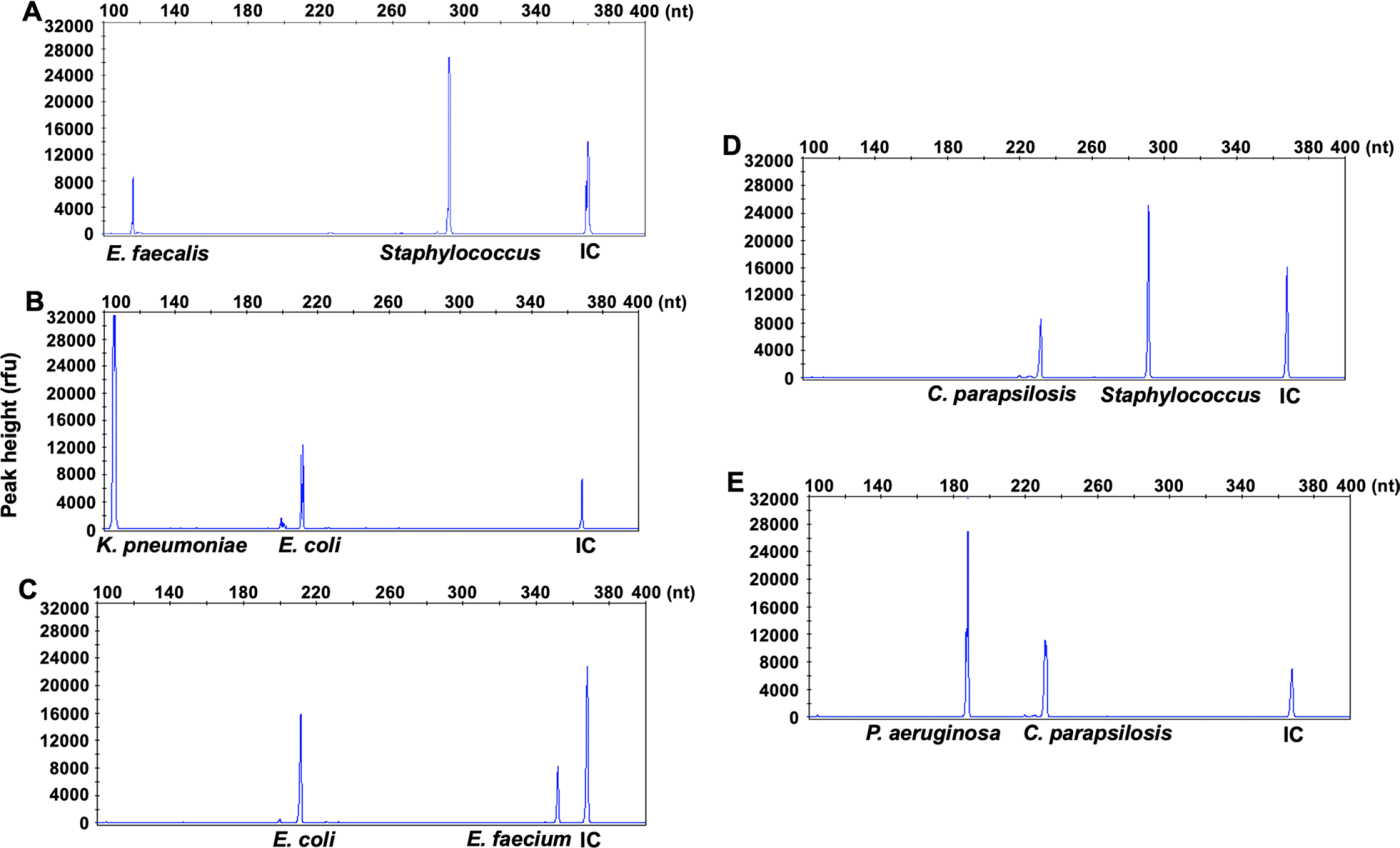
BSI-HMGS robustly detects individual pathogen in polymicrobial mixtures. Mixtures of plasmids corresponding to pathogens that commonly appear in clinical polymicrobial BSIs, including dual infections with two gram-positive bacteria (**A**), two gram-negative bacteria **(B**), gram-negative bacteria and gram-positive bacteria (**C**), gram-positive bacteria and fungi (**D**), and gram-negative bacteria and fungi (**E**), were detected using BSI-HMGS. Notably, a tenfold concentration difference was applied to the two plasmids in each mixture to verify the BSI-HMGS performance for detecting multiple infections with a distinct load difference. The IC peaks fluctuates though with the same concentration used, which may result from the systematic error caused during the nucleic acid extraction process, amplification, or capillary electrophoresis process, and the IC signal may also competitively affected by other targets in the amplification reaction system. HMGS: high-throughput multiplex genetic detection system; IC: internal control

To demonstrate the capability of BSI-HMGS to detect polymicrobial infections, five mixtures of two pathogen plasmids, which comprised the most common combinations detected in clinical practice for gram-negative bacteria, gram-positive bacteria and fungi, along with the IC were used. Moreover, considering the possibility of different loads of pathogens in polymicrobial infections, the interference from microbial DNA with high template quantities to the detection of low-abundance DNA targets was assessed by setting the concentrations of the two plasmids in each mixture at a tenfold difference (including *Staphylococcus*, 1.0 × 10^3^ copies/μL mixed with *E. faecalis*, 1.0 × 10^2^ copies/μL; *K. pneumoniae*, 1.0 × 10^3^ copies/μL mixed with *E. coli*, 1.0 × 10^2^ copies/μL; *E. coli*, 1.0 × 10^3^ copies/μL mixed with *E. faecium*, 1.0 × 10^2^ copies/μL; *C. parapsilosis*, 1.0 × 10^3^ copies/μL mixed with *Staphylococcus*, 1.0 × 10^2^ copies/μL; and *P. aeruginosa*, 1.0 × 10^3^ copies/μL mixed with *C. parapsilosis*, 1.0 × 10^2^ copies/μL). As shown in Fig. 4, specific amplification peaks of the mixed plasmids were observed, and the signal magnitudes of low-abundance templates generated in the BSI-HMGS assay remained high and reached over 6000 rfu. Thus, these results indicated that no mutual interference was seen at the concentration differences chosen for this experiment, excluding the possibility that the higher copy number target would outcompete a target sequence at a lower copy number within the ranges of templates used in our studies.

### Performance of BSI-HMGS combined with the pre-treatment protocol on blood cultures

To test the performance of BSI-HMGS on blood culture samples (*step (ii) and (iii) of the pre-treatment protocol were applied for treating the blood cultures*), 526 clinical blood culture samples were analysed. According to the reference results, 100 samples were negative and 426 were positive for the target pathogens of BSI-HMGS; of these, one case of double infection with *E. coli* and *S. maltophilia* was detected. No positive sample for *M. catarrhalis* was detected during the collection period. Notably, when analysing the detection results of the BSI-HMGS assay, the numbers of two-peak pathogens, including *S. aureus*, *S. agalactiae*, *S. pneumoniae*, and *S. pyogenes* were calculated individually, whereas the number of *Staphylococcus* represented the number of pathogens with a single *Staphylococcus* peak; the same method was applied for *Streptococcus*. In the current study, the median time to positivity in the Versa TREK system was 17.1 h (IQR: 15.3–19.5) and the following subculture and MALDI-TOF MS detection required additional 4–48 h for different pathogens. The time we needed to perform all the steps of BSI-HMGS and pre-treatment protocol from blood cultures was about 3 h, among which the concentration and inhibitor removal required about 0.5 h and identification with the BSI-HMGS assay required about 2.5 h. The BSI-HMGS assay results of the 526 samples were highly consistent with the reference results (Table 2). The double infection sample was also identified correctly by BSI-HMGS. The NPAs of BSI-HMGS detecting different target pathogens from blood culture samples were all 100.0% and the PPAs ranged from 87.5–100.0%. The results showed that 6 (1.1%) of the 526 blood culture samples were culture positive/BSI-HMGS negative, including one *K. pneumoniae*, one *A. baumannii*, one *S. epidermidis*, one *S. haemolyticus*, one *S. salivarius*, and one *C. albicans*. One case of *B. cepacia* infection was culture positive/MALDI TOF MS identified as *B. multivorans*/BSI-HMGS positive. Furthermore, dilution of nucleic acid extracts, mainly used to dilute the PCR inhibitors, was not needed before every successful identification. Therefore, these results confirmed the reliability and reproducibility of the BSI-HMGS assay for pathogen detection in blood culture samples.

**Table 2:** Performance evaluation of BSI-HMGS in the detection of clinical blood culture samples

Pathogens	HMGS	Reference results		PPA	NPA	OPA
		+	−			
*K. pneumoniae*	+	63	0	0.984	1.000	0.998
	−	1	462			
*B. cepacia*	+	1	0	1.000	1.000	1.000
	−	0	525			
*P. mirabilis*	+	3	0	1.000	1.000	1.000
	−	0	523			
*M. catarrhalis*	+	0	0	NA	NA	NA
	−	0	526			
*S. marcescens*	+	13	0	1.000	1.000	1.000
	−	0	513			
*H. influenzae*	+	1	0	1.000	1.000	1.000
	−	0	525			
*P. aeruginosa*	+	16	0	1.000	1.000	1.000
	−	0	510			
*E. cloacae* complex	+	9	0	1.000	1.000	1.000
	−	0	517			
*E. coli*	+	56	0	1.000	1.000	1.000
	−	0	470			
*A. baumannii*	+	14	0	0.933	1.000	0.998
	−	1	511			
*S. maltophilia*	+	10	0	1.000	1.000	1.000
	−	0	516			
*S. enterica*	+	2	0	1.000	1.000	1.000
	−	0	524			
*Staphylococcus*	+	56	0	0.966	1.000	0.996
	−	2	468			
*S. aureus*	+	36	0	1.000	1.000	1.000
	−	0	490			
*Streptococcus*	+	7	0	0.875	1.000	0.998
	−	1	516			
*S. pyogenes*	+	1	0	1.000	1.000	1.000
	−	0	525			
*S. agalactiae*	+	4	0	1.000	1.000	1.000
	−	0	522			
*S. pneumoniae*	+	2	0	1.000	1.000	1.000
	−	0	524			
*E. faecalis*	+	15	0	1.000	1.000	1.000
	−	0	511			
*E. faecium*	+	28	0	1.000	1.000	1.000
	−	0	498			
*C.albicans*	+	40	0	0.976	1.000	0.998
	−	1	485			
*C. tropicalis*	+	8	0	1.000	1.000	1.000
	−	0	518			
*C. parapsilosis*	+	19	0	1.000	1.000	1.000
	−	0	507			
*C. glabrata*	+	17	0	1.000	1.000	1.000
	−	0	509			

The number of *Staphylococcus* and *Streptococcus* represent the number of pathogens with a single *Staphylococcus* and *Streptococcus* peak, respectively, excluding the two-peak pathogens. HMGS: high-throughput multiplex genetic detection system; PPA: positive percent agreement; NPA: negative percent agreement; OPA: overall percent agreement; NA: not applicable

### Performance of BSI-HMGS combined with the pre-treatment protocol on whole blood samples

The performance of BSI-HMGS combined with the pre-analytical treatment protocol on whole/simulated whole blood samples was then evaluated. In total, 214 clinical samples were obtained from the enrolled patients, then treated by the pre-analytical treatment protocol and detected using the BSI-HMGS. According to the final reference results, 25 (11.7%) samples were positive for 13 target pathogens in BSI-HMGS whereas 189 (88.3%) samples were negative (Table 3). Compared to the reference results, the NPAs of BSI-HMGS for the detection of these pre-treated samples were 100.0%, and the PPAs ranged from 66.7% to 100.0%, among which 10 out of 13 pathogens were 100.0%. Of the 214 clinical pre-treated samples, 3 (1.4%) were culture positive/BSI-HMGS negative, including one *K. pneumoniae*, one *S. hominis* and one *C. albicans*. The OPAs of BSI-HMGS for detecting these pre-treated clinical samples were greater than 99.5%. In this study, the time we needed to perform all the steps of BSI-HMGS and pre-treatment protocol from whole blood was about 8 h, among which the pre-analytical incubation, concentration, and inhibitor removal required about 5.5 h and identification with the BSI-HMGS assay required about 2.5 h.

**Table 3:** Performance evaluation of BSI-HMGS combined with the pre-treatment protocol on whole blood samples

Pathogens	HMGS	Reference results		PPA	NPA	OPA
		+	−			
*K. pneumoniae*	+	3	0	0.750	1.000	0.995
	−	1	210			
*P. aeruginosa*	+	2	0	1.000	1.000	1.000
	−	0	212			
*E. cloacae* complex	+	1	0	1.000	1.000	1.000
	−	0	213			
*E. coli*	+	3	0	1.000	1.000	1.000
	−	0	211			
*A. baumannii*	+	1	0	1.000	1.000	1.000
	−	0	213			
*S. maltophilia*	+	1	0	1.000	1.000	1.000
	−	0	213			
*Staphylococcus*	+	2	0	0.667	1.000	0.995
	−	1	211			
*S. aureus*	+	2	0	1.000	1.000	1.000
	−	0	212			
*Streptococcus*	+	2	0	1.000	1.000	1.000
	−	0	212			
*E. faecalis*	+	1	0	1.000	1.000	1.000
	−	0	213			
*E. faecium*	+	1	0	1.000	1.000	1.000
	−	0	213			
*C.albicans*	+	2	0	0.667	1.000	0.995
	−	1	211			
*C. glabrata*	+	1	0	1.000	1.000	1.000
	−	0	213			

The number of *Staphylococcus* and *Streptococcus* represent the number of pathogens with a single *Staphylococcus* and *Streptococcus* peak, respectively, excluding the two-peak pathogens. HMGS: high-throughput multiplex genetic detection system; PPA: positive percent agreement; NPA: negative percent agreement; OPA: overall percent agreement

Given that the number of these pre-treated clinical whole blood samples, especially the positive samples, was relatively small, some positive whole blood samples were simulated to further validate the pre-treatment protocol and BSI-HMGS. In total, 720 samples were simulated with 30 samples for each pathogen. The results showed that the NPAs of BSI-HMGS for detecting these simulated pre-treated samples were 100.0% and the PPAs ranged from 80.0% to 100.0%, with 16 out of 24 pathogens being greater than 90.0%, and all OPAs were greater than 99.2% (Table 4). Further, of the 720 simulated samples, 59 (8.2%) were culture positive/BSI-HMGS negative, and the PPAs of *P. mirabilis*, *H. influenzae*, *P. aeruginosa*, *S. maltophilia, S. agalactiae*, *C.albicans*, *C. tropicalis* and *C. glabrata* were below 90%. Moreover, in the simulation procedures, the amount of pathogen used is equivalent to that in 10 mL positive whole blood sample with a concentration of 10 CFU/mL in clinical practice, further indicating that the pre-treatment protocol combined with BSI-HMGS could detect clinical samples sensitively. Collectively, these data demonstrated that the combined BSI-HMGS and pre-treatment protocol is efficient for detecting BSI pathogens in whole blood samples.

**Table 4:** Performance evaluation of BSI-HMGS combined with the pre-treatment protocol on simulated whole blood samples

Pathogens	HMGS	Reference results		PPA	NPA	OPA
		+	−			
*K. pneumoniae*	+	29	0	0.967	1.000	0.999
	−	1	690			
*B. cepacia*	+	29	0	0.967	1.000	0.999
	−	1	690			
*P. mirabilis*	+	26	0	0.867	1.000	0.994
	−	4	690			
*M. catarrhalis*	+	28	0	0.933	1.000	0.997
	−	2	690			
*S. marcescens*	+	27	0	0.900	1.000	0.996
	−	3	690			
*H. influenzae*	+	26	0	0.867	1.000	0.994
	−	4	690			
*P. aeruginosa*	+	25	0	0.833	1.000	0.993
	−	5	690			
*E. cloacae* complex	+	30	0	1.000	1.000	1.000
	−	0	690			
*E. coli*	+	30	0	1.000	1.000	1.000
	−	0	690			
*A. baumannii*	+	29	0	0.967	1.000	0.999
	−	1	690			
*S. maltophilia*	+	24	0	0.800	1.000	0.992
	−	6	690			
*S. enterica*	+	28	0	0.933	1.000	0.997
	−	2	690			
*Staphylococcus*	+	27	0	0.900	1.000	0.996
	−	3	690			
*S. aureus*	+	29	0	0.967	1.000	0.999
	−	1	690			
*Streptococcus*	+	29	0	0.967	1.000	0.999
	-	1	690			
*S. pyogenes*	+	29	0	0.967	1.000	0.999
	−	1	690			
*S. agalactiae*	+	25	0	0.833	1.000	0.993
	−	5	690			
*S. pneumoniae*	+	30	0	1.000	1.000	1.000
	−	0	690			
*E. faecalis*	+	30	0	1.000	1.000	1.000
	-	0	690			
*E. faecium*	+	29	0	0.967	1.000	0.999
	−	1	690			
*C.albicans*	+	26	0	0.867	1.000	0.994
	−	4	690			
*C. tropicalis*	+	24	0	0.800	1.000	0.992
	−	6	690			
*C. parapsilosis*	+	27	0	0.900	1.000	0.996
	−	3	690			
*C. glabrata*	+	25	0	0.833	1.000	0.993
	−	5	690			

The number of *Staphylococcus* and *Streptococcus* represent the number of pathogens with a single *Staphylococcus* and *Streptococcus* peak, respectively, excluding the two-peak pathogens. HMGS: high-throughput multiplex genetic detection system; PPA: positive percent agreement; NPA: negative percent agreement; OPA: overall percent agreement

## Discussion

Bloodstream infections continue to be a leading cause of death in hospitalized patients, and early and targeted antimicrobial intervention is the principal determinant of clinical outcome [23]. Therefore, accurate and timely etiologic diagnosis of BSIs is critical to control disease progression. However, the current diagnostic approaches fail to provide actionable information within a clinically viable time frame because they rely on time-consuming blood cultures [24]. There are several molecular diagnostic assays based on sequencing, fluorescence-based PCR detection, or microarray commercially available for the detection of BSIs pathogens from blood culture bottles and directly from whole blood, some of which are unaffected by PCR inhibitors that may be introduced by the patient’s blood sample, nor the components in the blood culture bottles [25, 26]. While these methods have matured in the diagnostics of BSI, room for further exploration remains. Such as sequencing requires advanced technology for instruments and operators, and is unavailable in a majority of health centres, especially in underdeveloped countries with limited medical resources; the PCR-based methods for pathogen detection directly in whole blood are highly influenced by input sample volume and the method used for the enrichment of microbial DNA [27, 28]. In this study, we developed an integrated pre-treatment protocol, capable of pathogen enrichment by short incubation and concentration, and removal of inhibitors within 5.5 h prior to molecular analysis, followed by a multiplex genetic detection system, BSI-HMGS, capable of simultaneous detection of the clinically most prevalent BSI pathogens. The combined pre-treatment protocol and the BSI-HMGS successfully detects microbes in whole blood samples, which may not meet the demand for clinical detection using normal PCR-based methods due to the existence of PCR inhibitors and or low pathogen loads.

An important difference between blood samples and other clinical samples is that the etiologic diagnosis of BSIs using PCR-based methods might be inhibited by non-target DNA, heparin, and immunoglobulins in human blood [19, 20]. The existence of PCR inhibitors in the matrix components of current predominantly used blood culture bottles was verified, which persisted even after nucleic acid extraction. Moreover, the pathogens loads in whole blood from patients with sepsis may be even less than 10 CFU/mL, which is below the detection limit of common PCR-based methods [16]. This also accords with the preliminary experiments, which showed that the PCR-based method failed to identify pathogens from whole blood samples directly. Thus, proper pre-analytical enrichment of pathogens and depletion of potential PCR inhibitors is necessary for accurate pathogen identification.

Commercial blood culture bottles with nutrient-rich conditions and counteracting effects of antimicrobial agents are suitable for the enrichment of pathogens in blood samples due to the common antimicrobial treatments initiated before blood sampling. The short incubation facilitates obtaining more templates for subsequent detection. Next, the centrifugation adopting a serum separation tube (SST) can enrich pathogens from up to 5–10 mL samples, yielding an equivalent of 5–10 mL sample input volume per PCR, and the removal of the supernatant after centrifugation can also reduce potentially interfering factors efficiently, through which the pathogen load assayed was increased directly and the pathogen was preliminarily purified. This approach had been successfully applied for the enrichment of pathogens from blood culture samples for subsequent identification by MALDI-TOF MS, which proved to be able to capture bacteria effectively out of such a substrate and no side effect was reported [29–31]. Finally, despite the depletion of factors that interfere with PCR, inhibition was still observed after the above process, which resulted in relatively low peaks in the detection of positive samples and even false negative results. In addition, none of the extraction kits based on distinct principles used in this study was effective for removing the inhibitors. To minimize such interference, lysis and washing steps for inhibitors were conducted in the pre-treatment protocol, after which the inhibition decreased obviously (Fig. 1). The lysis buffer containing 10% SDS used in this study has been applied in the purification of carbapenemase-producing *Enterobacteriales* from blood cultures for subsequent identification and classification [32], and its effect on other target pathogens in the current panel was also evaluated to rule out that the 10% SDS might cause fragmentation of bacterial/fungal cells. No apparent fragmentation was observed through the current validation method. As is often observed in laboratories, in the absence of bacterial/fungal cell wall degradation enzymes such as lysozyme or lysostaphin, it is difficult to extract bacterial/fungal nucleic acids in the 10% SDS solution. In this study, the washing step removed most residual SDS; moreover, the cells were broken by vortexing with glass beads in the nucleic acid extraction step, which can significantly attenuate the effect of SDS. In summary, the utility of this integrated pre-treatment protocol is promising for the early identification of BSIs pathogens.

The target pathogens were selected based on the data from epidemiological investigations. The five most prevalent species causing BSI in China were *E. coli*, *K. pneumoniae*, *P. aeruginosa*, *A. baumannii*, and *S. aureus*, which accounted for more than 50% of BSIs [33]. In another epidemiologic report on BSIs from 45 nations, these five species plus *E. cloacae*, *E. faecalis*, *E. faecium*, *S. epidermidis* and *S. agalactiae* accounted for more than 70% of cases [2]. Meanwhile, contamination of blood cultures occurs frequently in the clinic. In this study, to minimize the potential for contamination, UNG was used in the amplification process to avoid amplicon contamination, and the contaminating micro-organisms with relatively lower loads usually cannot meet the test demand of PCR-based method without a long-time incubation. In short, the potential contamination cannot be completely ruled out due to the low LOD of a molecular method, but the risk of contamination is low when all necessary precautions are taken.

Performance assessment using blood cultures and whole blood samples highlighted the robustness of the pre-treatment protocol combined with BSI-HMGS. For blood culture samples, high PPAs and NPAs were obtained for all target pathogens compared with the reference results. While in comparison with the routine methods on blood cultures, the current method does not present many advantage in terms of time-saving due to that the time to positivity is still the most time-consuming process, and the relatively much more hands-on manipulation impedes its necessity of application in the routine clinical practice. Thus, the BSI-HMGS may serve as a temporary method in the identification of positive blood cultures, and enable physicians to confirm the presence of suspected pathogens and adjust the empirical antimicrobial regimen early. For the whole/simulated whole blood samples, along with the pre-treatment protocol that enhances the pathogen loads, the BSI-HMGS showed PPAs of ≥ 80.0% in most cases and high NPAs of 100.0%. The discrepant results mainly showed as reference result-positive/BSI-HMGS-negative, among which the false negative results were mainly found in the detection of *P. mirabilis*, *H. influenzae*, *P. aeruginosa*, *S. maltophilia, S. agalactiae*, *C.albicans*, *C. tropicalis* and *C. glabrata*. This may be related to that these bacteria/fungi PCRs are less sensitive than the others for the detection of low bacterial/fungal loads, and or the methodology used to enrich cells and isolate microbial DNA may have various effects on different micro-organisms. Further, more false negative results were found in simulated whole blood samples (8.2%) than clinical whole blood samples (1.4%), and the small number of positive clinical whole blood samples may contribute. Also, for the preparation of simulated whole blood samples, the detection results using human blood would be more applicable in clinical settings, and the use of sheep blood in the current study may have some potential impact on the analytical data, and future studies are needed for specifically addressing this point. Moreover, the detection of *Candida* species in the simulated samples appeared relatively poor. The *Candida* positive samples spiked with laboratory-grown *Candida* species rather than from patients with true Candidemia may have specially influenced the test performance, which might result from changes in cell wall characteristics [34]. Thus, methods for the pre-treatment of specific pathogens can be investigated in further studies.

For the simulated whole blood samples, quantified bacterial/fungal suspensions were processed by all pre-analytical steps and then tested by the BSI-HMG, and the amount of pathogen is equivalent to that in 10 mL clinical blood sample, which is the suggested volume used for blood culture in the *Manual of Clinical Microbiology* (Chapter 4, 12th Edition), with a concentration of 10 CFU/mL. Thus, the detection results of the simulated samples indicate that the combined pre-treatment protocol and BSI-HMGS possessed a lower detection limit to approximately 10 CFU/mL for clinical samples. Most importantly, the current method provided robust results while simultaneously reducing the turnaround time to about 8 h, and BSI-HMGS can be performed in general laboratories with good experience in bio-molecular technologies to apply all the procedures described in the study, which can facilitate clinicians of more timely and precise treatment strategies.

## Conclusions

In summary, the current study presents an integrated pre-treatment protocol that enables pathogen enrichment and inhibitor removal from whole blood samples, and establishes a rapid and robust multiplex pathogen identification system, BSI-HMGS, which allows the detection of 24 targets and covers the most common BSI pathogens. Given that in most laboratories, more than 80% of the blood cultures are negative[35, 36], which may result in a lot of hands-on work for the few positive blood cultures, BSI-HMGS and pre-treatment protocol can be performed from positive or negative blood cultures and from whole blood only of critically ill patients according to particular request of the clinicians.

## Supplementary Information

**Additional file 1**. Additional figures and legends.

## Data Availability

The datasets supporting the conclusions of this article are included within the article and its additional files.

## Abbreviations

BSI: Blood stream infection
HMGS: High-throughput multiplex genetic detection system
PCR: Polymerase chain reaction
CFU: Colony forming units
MCF: McFarland turbidity unit
MALDI-TOF MS: Matrix-Assisted Laser Desorption Ionization time-of-flight mass spectrometry
SST: Serum separation tube
IC: Internal control
UNG: Uracil-*N*-glycosylase
LOD: Limit of detection
OPA: Overall percent agreement
PPA: Positive percent agreement
NPA: Negative percent agreement
