# Supplementary material for: Rapid and precise identification of bloodstream infections using a pre-treatment protocol combined with high-throughput multiplex genetic detection system
Source: BMC Infect Dis. 2022 Nov 8;22:823. doi: 10.1186/s12879-022-07793-6 (PMC9644494; doi:10.1186/s12879-022-07793-6)
Supplement: Supplementary file 1 — Additional file 1. Additional figures and legends. [file 12879_2022_7793_MOESM1_ESM.pdf]

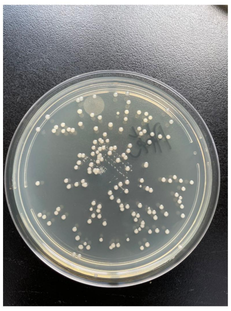
K. pneumoniae

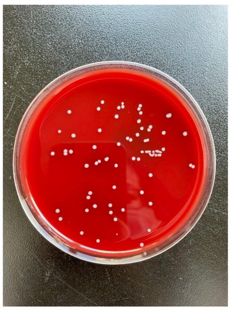
E.faecalis

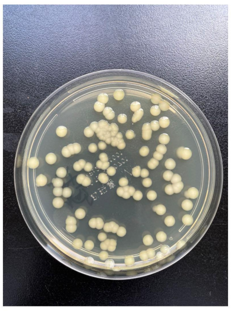
B.cepacia

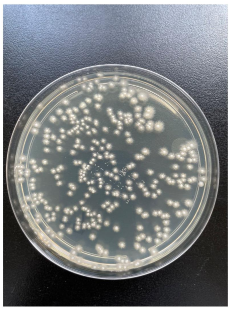
C.albicans

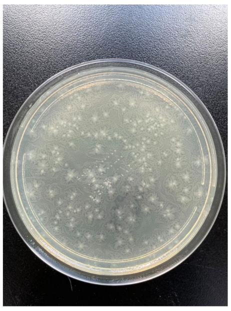
P.mirabilis

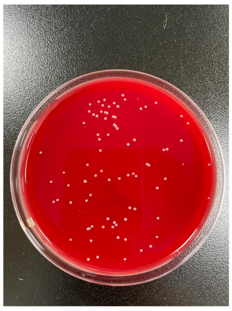
M.catarrhalis

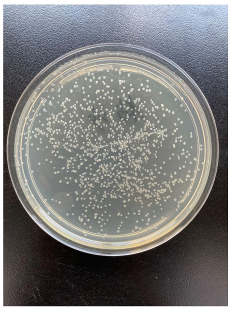
S.marcescens

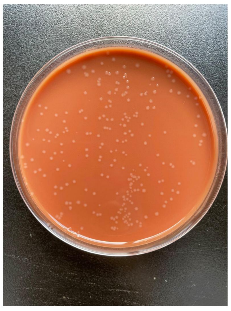
H.influenzae

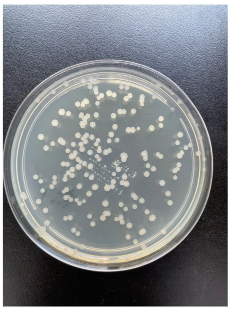
C.tropicalis

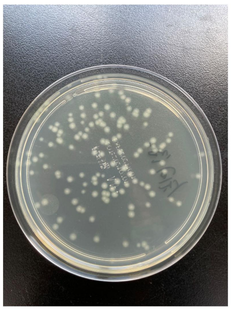
P.aeruginosa

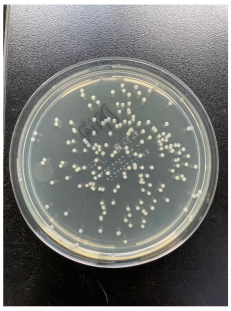
E. cloacae complex

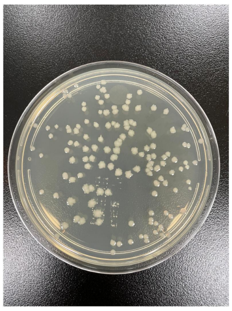
E. coli

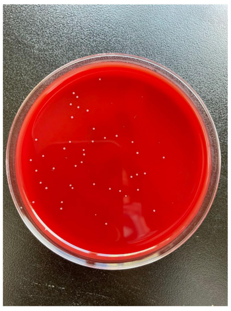
S. salivarius

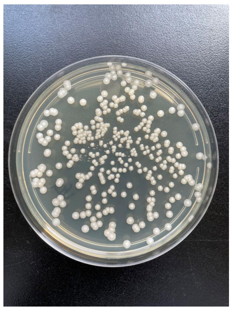
C. parapsilosis

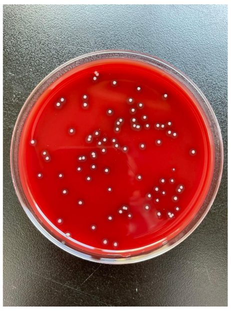
S. pyogenes

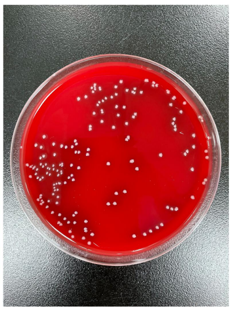
S. agalactiae

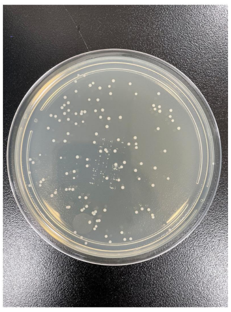
A. baumannii

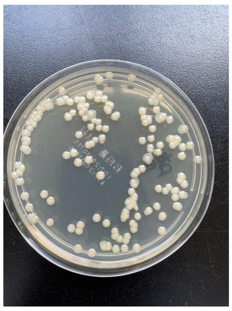
S. maltophilia

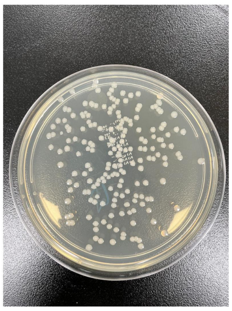
S. enteritidis

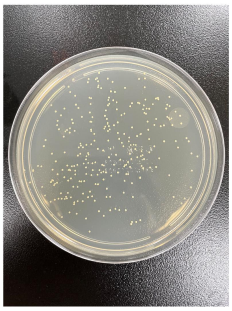
S.hominis

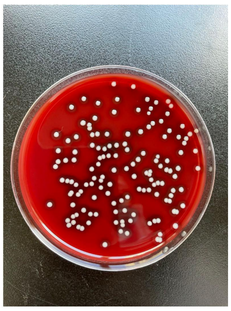
S. aureus

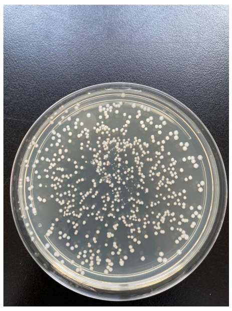
C. glabrata

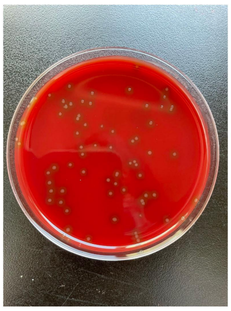
S. pneumoniae

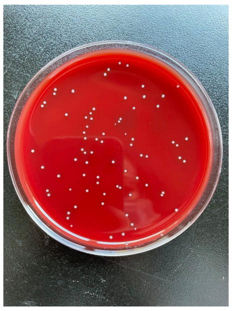
E. faecium

Figure S1. The countable colonies of 24 target organisms used for quantifications of corresponding bacterial/fungal suspensions

The bacterial/fungal suspensions with a turbidity of 0.5 McFarland turbidity unit (MCF) were serially diluted to the desired concentrations and incubated on appropriate solid culture mediums until colony-forming units (CFU) were visible. The bacterial suspension was totally diluted about 1.0×10^5 fold and the fungal suspension about 1.0×10^3 fold. Three replicates were made for each organism. Accurate CFU/mL of each 0.5 MCF suspension was determined by plate count.

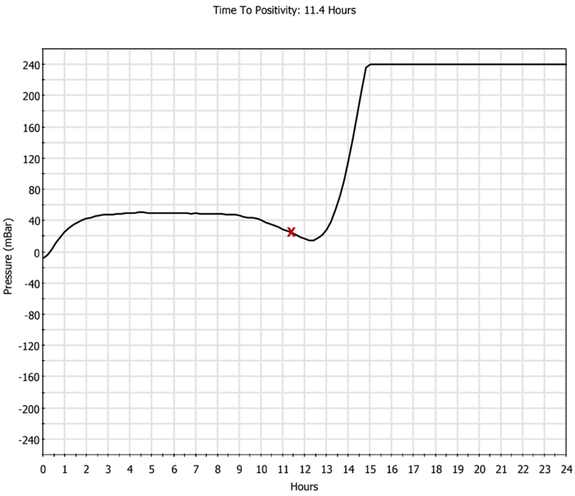

K. pneumoniae

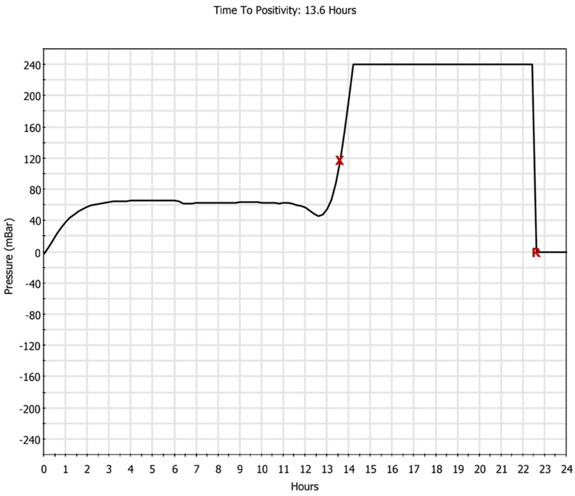

E.faecalis

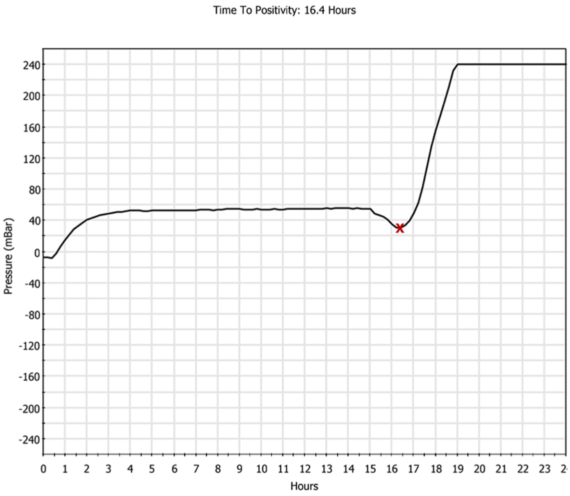

B.cepacia

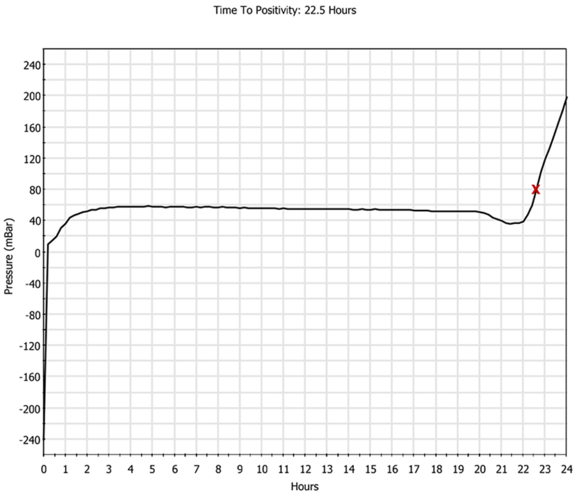

C.albicans

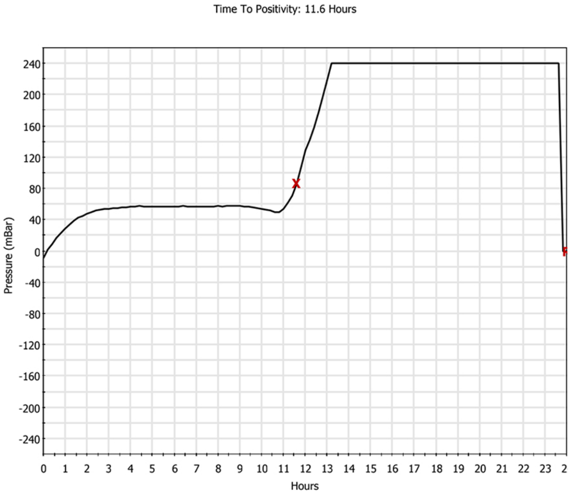

P.mirabilis

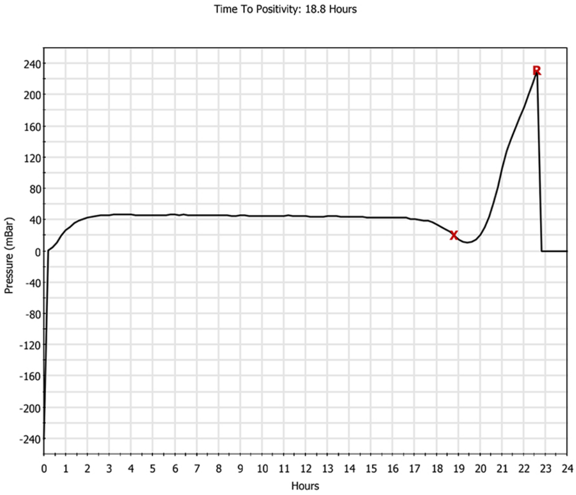

M.catarrhalis

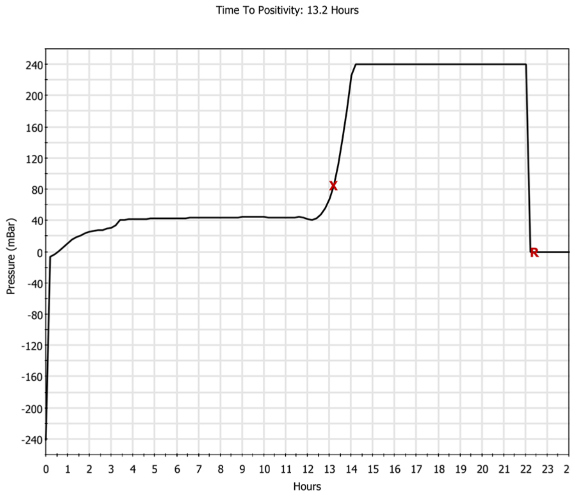

S.marcescens

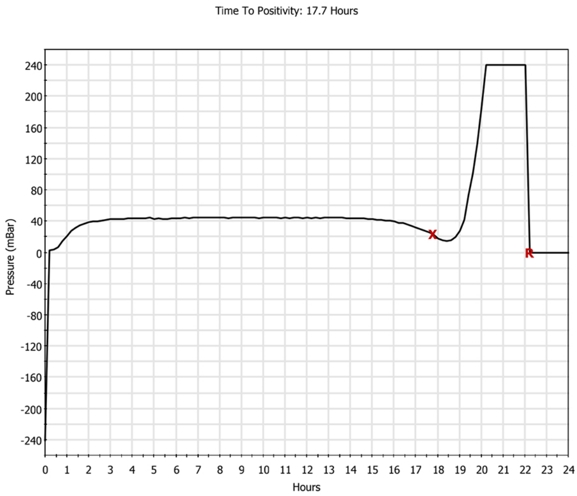

H.influenzae

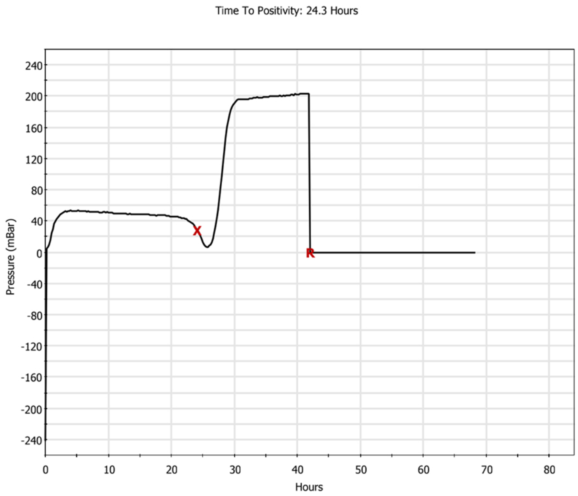

C.tropicalis

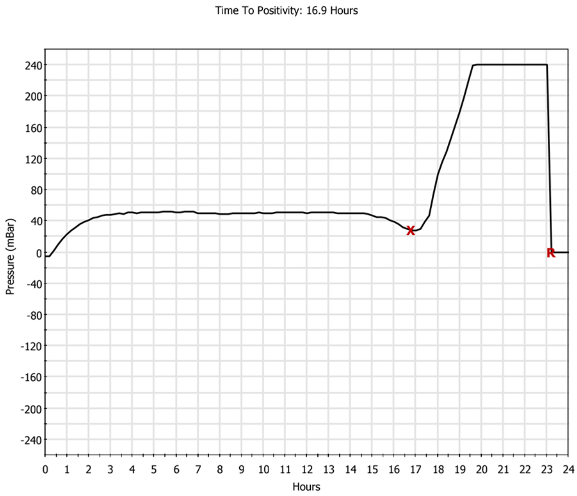

P.aeruginosa

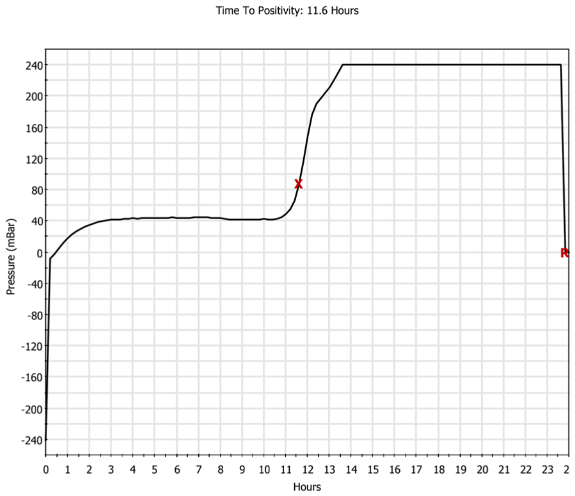

E. cloacae

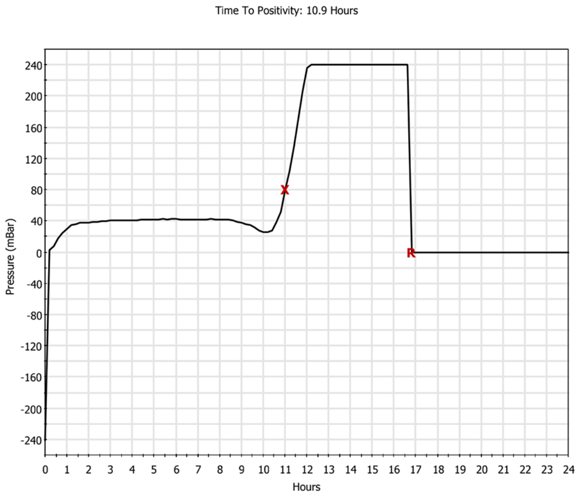

E. coli

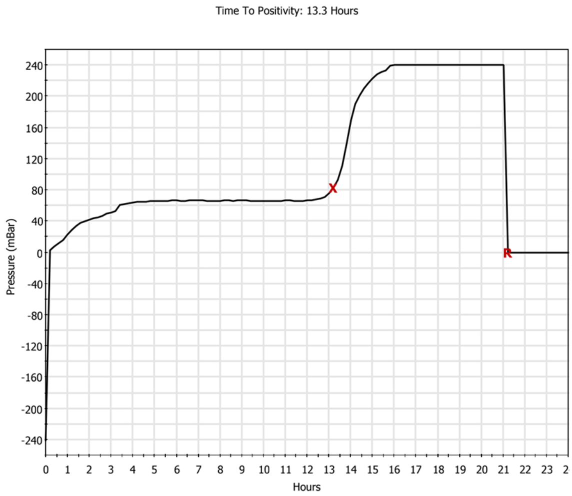

S. salivarius

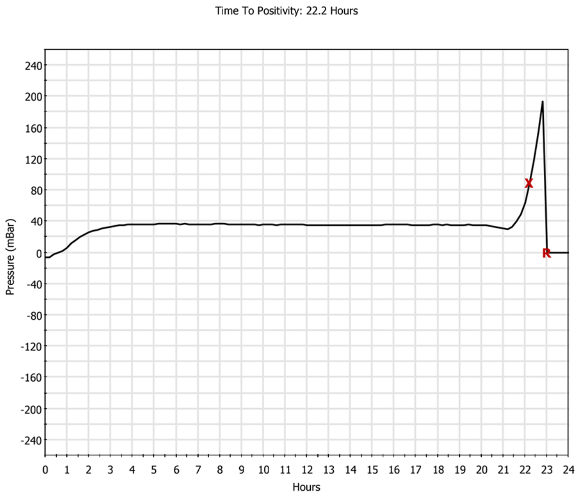

C. parapsilosis

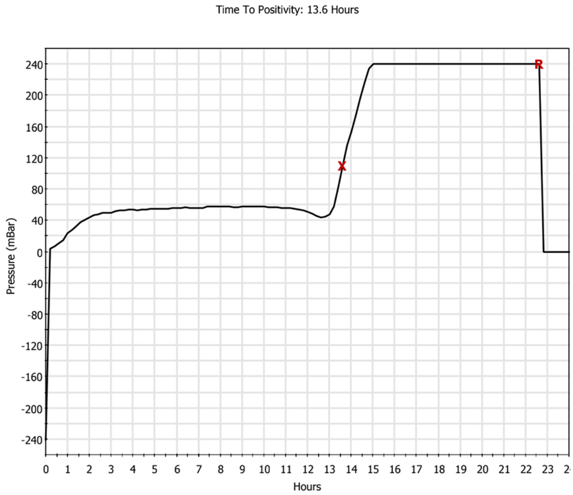

S. pyogenes

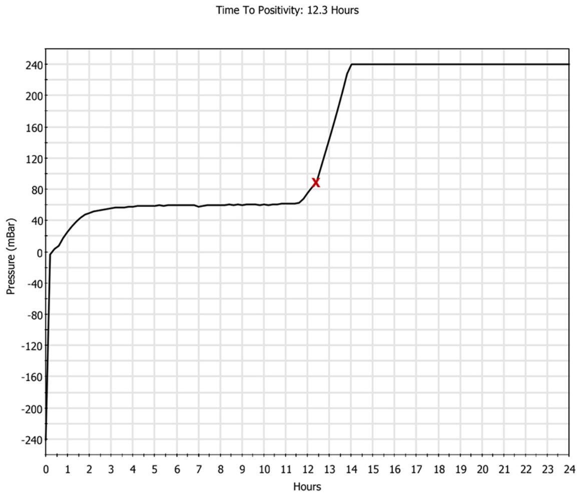

S. agalactiae

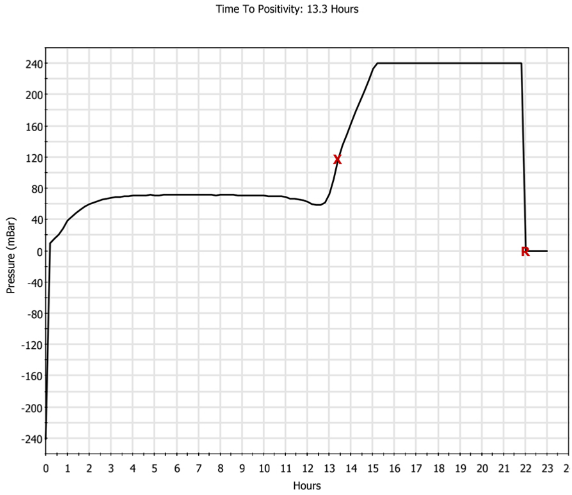

A. baumannii

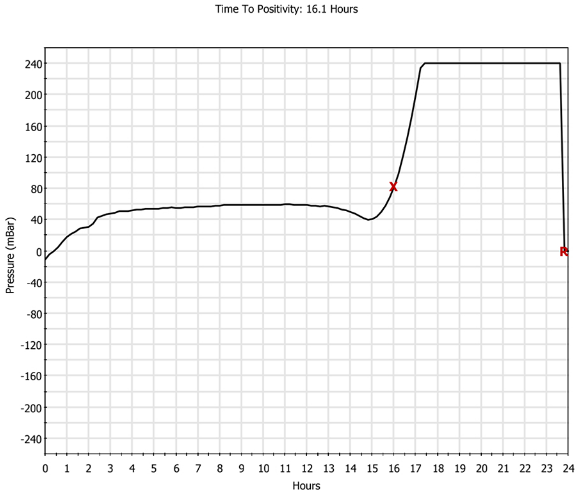

S. maltophilia

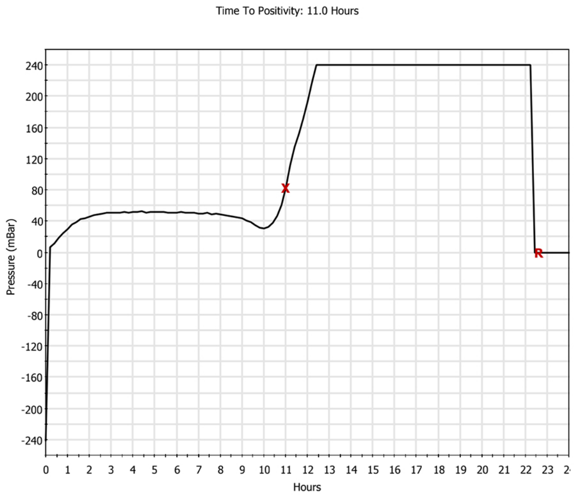

S. enteritidis

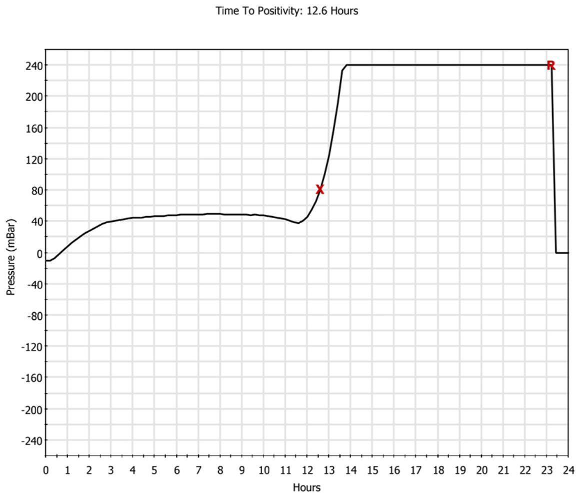

S.hominis

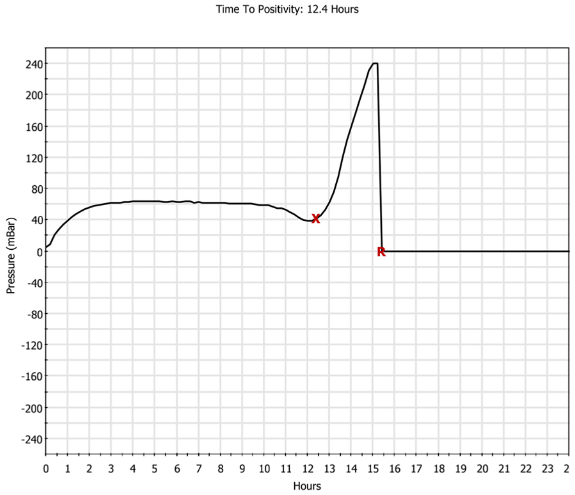

S. aureus

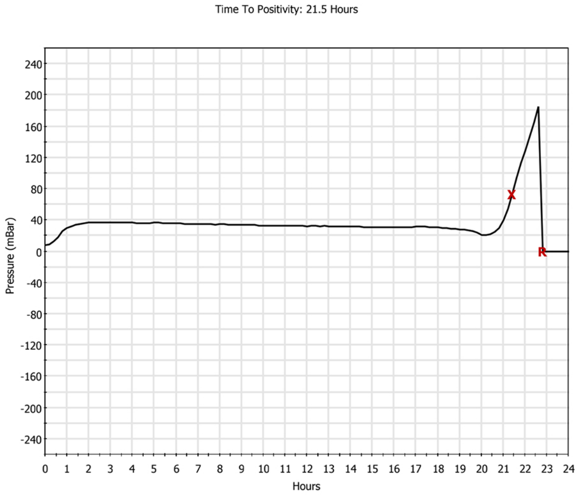

C. glabrata

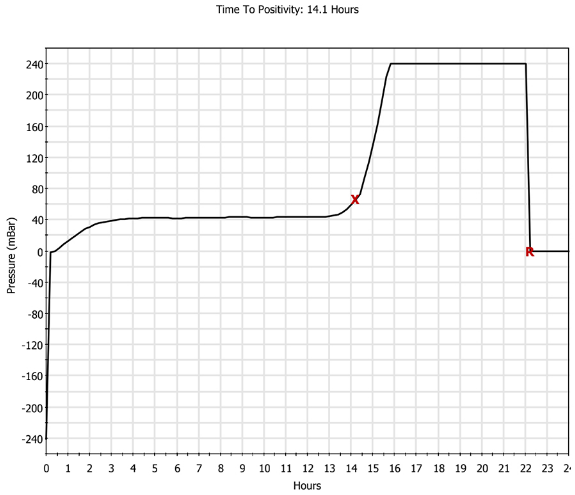

S. pneumoniae

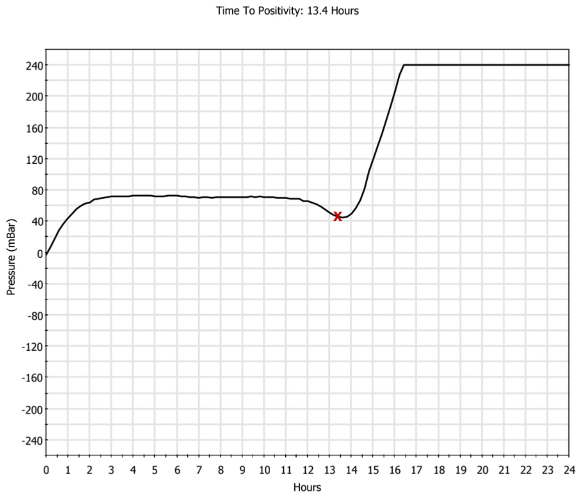

E. faecium

Figure S2. The original concentration–response curves for the bottles injected with 10 mL of 10 CFU/mL spiked blood samples from the Versa TREK system

The 24 concentration–response curves corresponding to all the target organisms for the bottles injected with 10 mL of 10 CFU/mL spiked blood samples from the Versa TREK were shown. X-axis represents the time of incubation and Y-axis represents the pressure which will change over the growth of the organism in the bottle. The “red X” in each panel indicates the point of reporting a positive result from the system and the “red R” indicates the point that the bottle was taken off from the system. Furthermore, in the preliminary study, other concentrations of spiked blood samples including 20 CFU/mL, 5 CFU/mL and 1 CFU/mL were also investigated. Compared with 10 CFU/mL , the lower concentration can also provide similar results to clinical samples but unstable, thus 10 CFU/mL spiked blood samples was used for simulation.

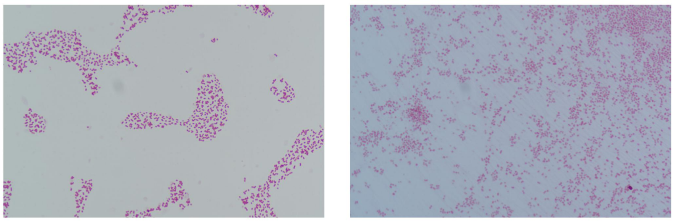

K. pneumoniae

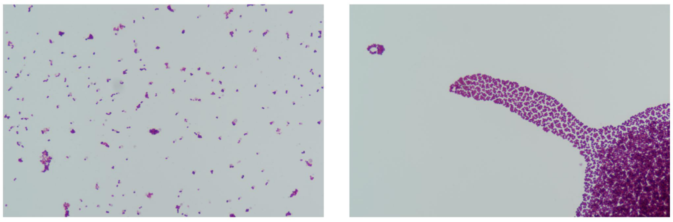

E.faecalis

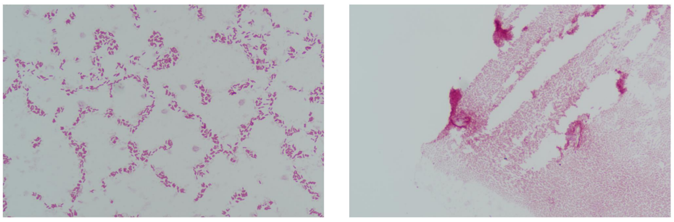

B.cepacia

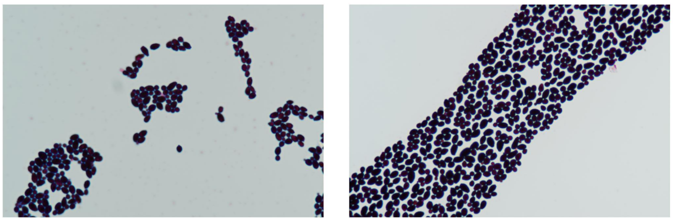

C.albicans

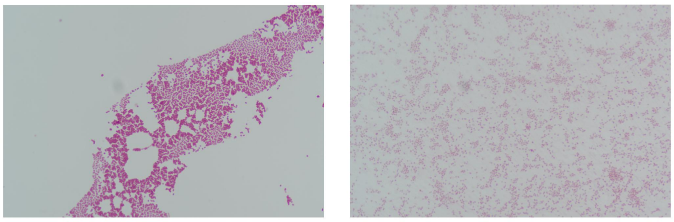

P.mirabilis

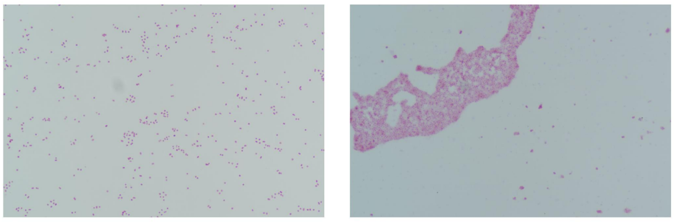

M.catarrhalis

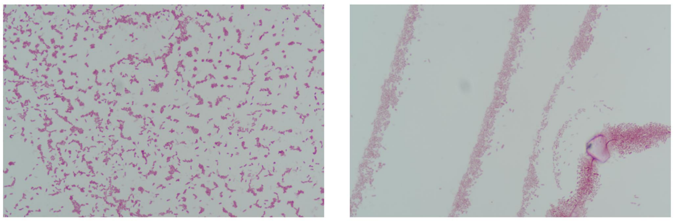

S.marcescens

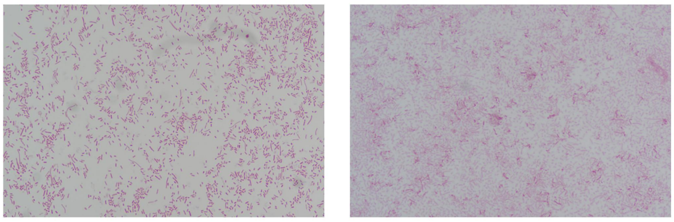

H.influenzae

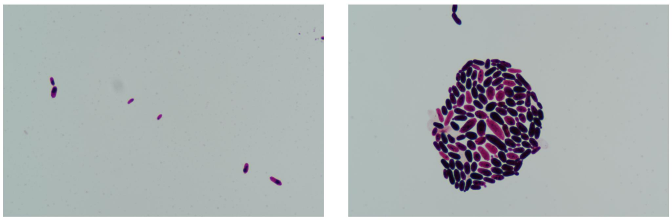

C.tropicalis

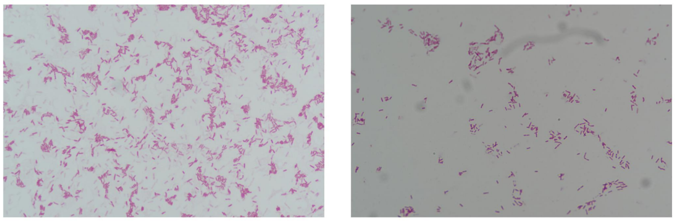

P.aeruginosa

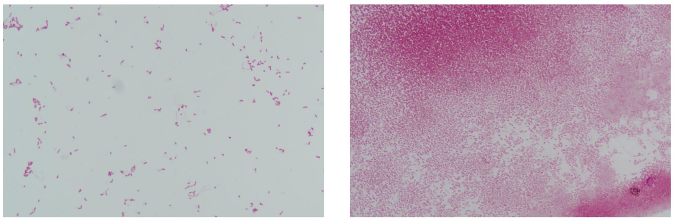

***E. cloacae* complex**

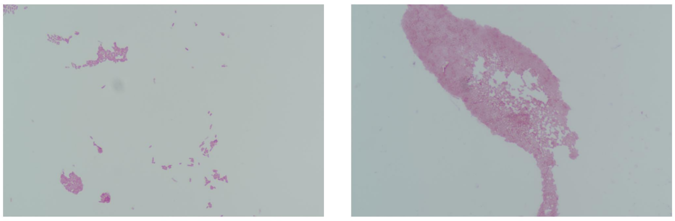

E. coli

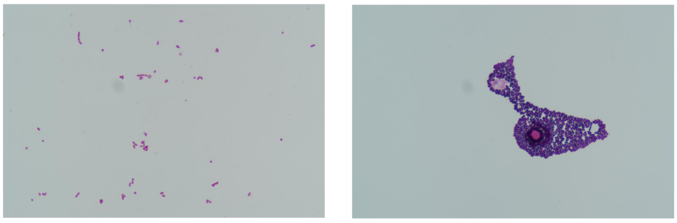

S. salivarius

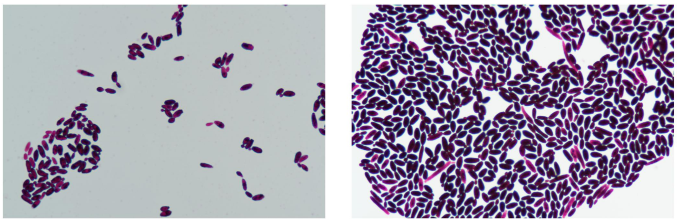

C. parapsilosis

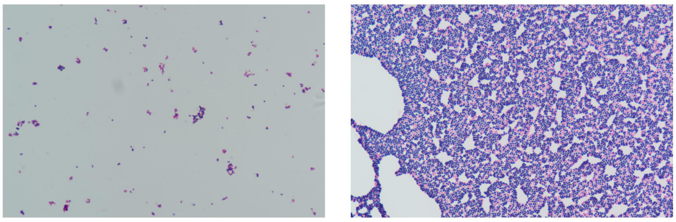

S. pyogenes

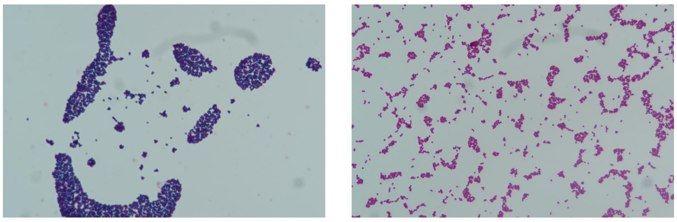

S. agalactiae

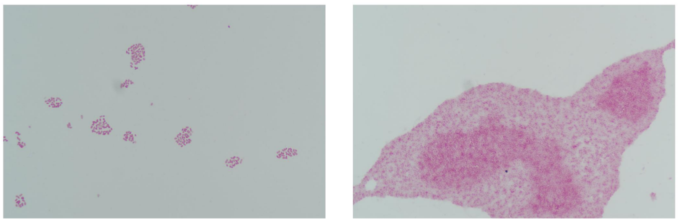

A. baumannii

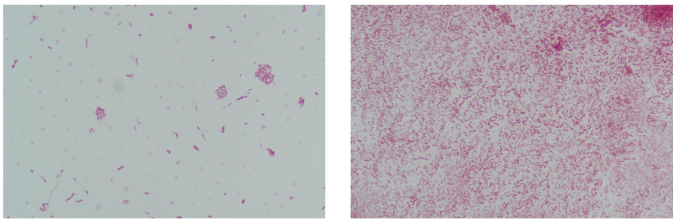

S. maltophilia

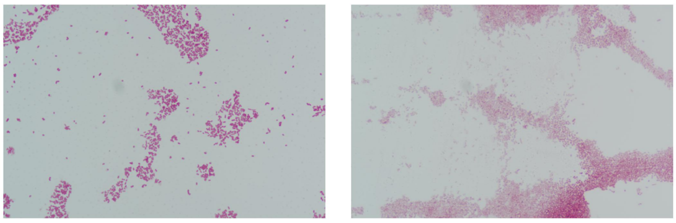

S. enteritidis

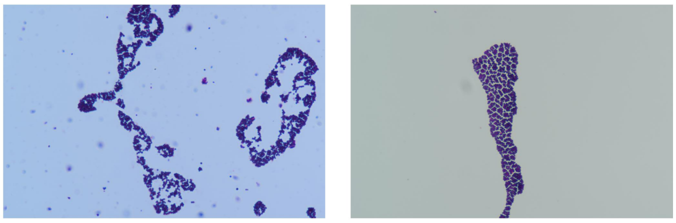

S.hominis

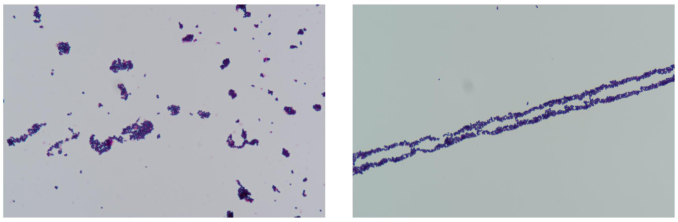

S. aureus

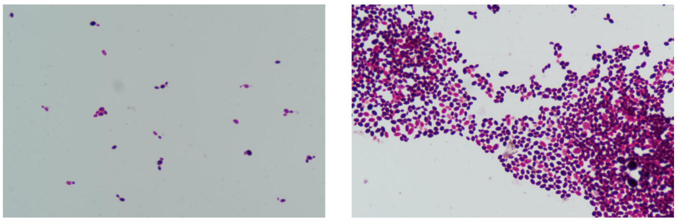

C. glabrata

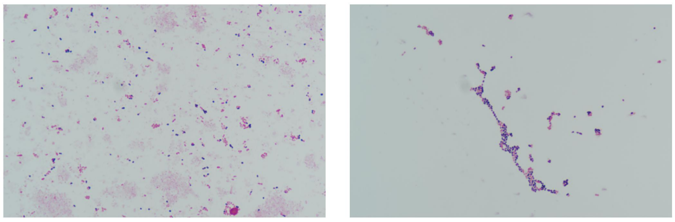

S. pneumoniae

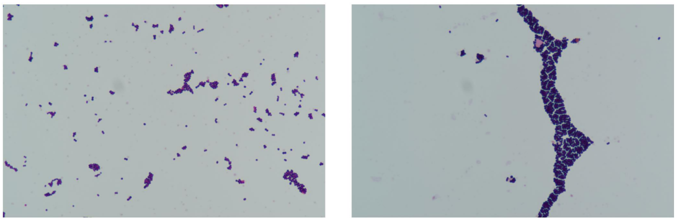

E. faecium

Figure S3. The effect of 10% sodium dodecyl sulphate (SDS) treatment to 24 target organisms in this study

To explore the effect of 10% SDS treatment on different target organisms, each organism suspension was treated with the lysis buffer (containing 10% SDS) and 0.45% NaCl in the same way as performed in the study, respectively, then the sediment was examined by smear microscopy. As shown in this figure, the image on the left of each organism shows the one treated by 0.45% NaCl and the right by lysis buffer, and compared with the control cells, some organisms treated by lysis buffer have slight morphological alterations which mainly present as increased aggregation. No obvious other morphological alteration was observed.

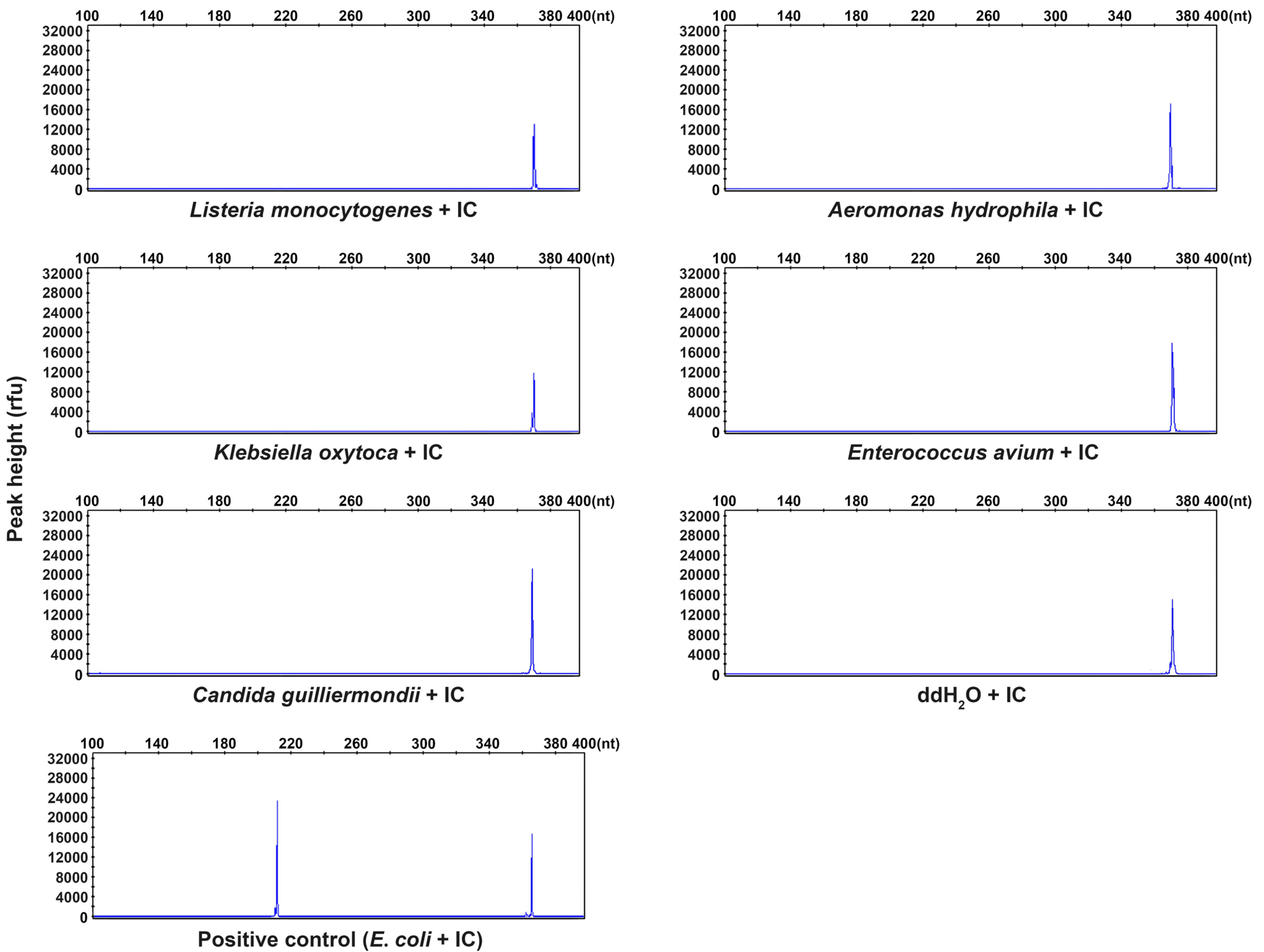

Peak height (rfu)
100 140 180 220 260 300 340 380 400(nt)
32000 28000 24000 20000 16000 12000 8000 4000 0
Listeria monocytogenes + IC
Aeromonas hydrophila + IC
Klebsiella oxytoca + IC
Enterococcus avium + IC
Candida guilliermondii + IC
ddH2O + IC
Positive control (E. coli + IC)

Figure S4. No non-specific amplification was observed when detecting pathogens not included in the targets of BSI-HMGS

Five pathogens that have been detected in BSIs but not included in BSI-HMGS, including *Listeria monocytogenes*, *Aeromonas hydrophila*, *Klebsiella oxytoca*, *Enterococcus avium* and *Candida guilliermondii* suspensions each at a concentration of approximately 1.0×10^5 CFU/mL, and ddH_2O were tested to rule out any non-specific amplification. No amplification except for IC was observed. *E. coli* was also detected as a positive control. Abbreviation: IC, internal control; HMGS, high-throughput multiplex genetic detection system.

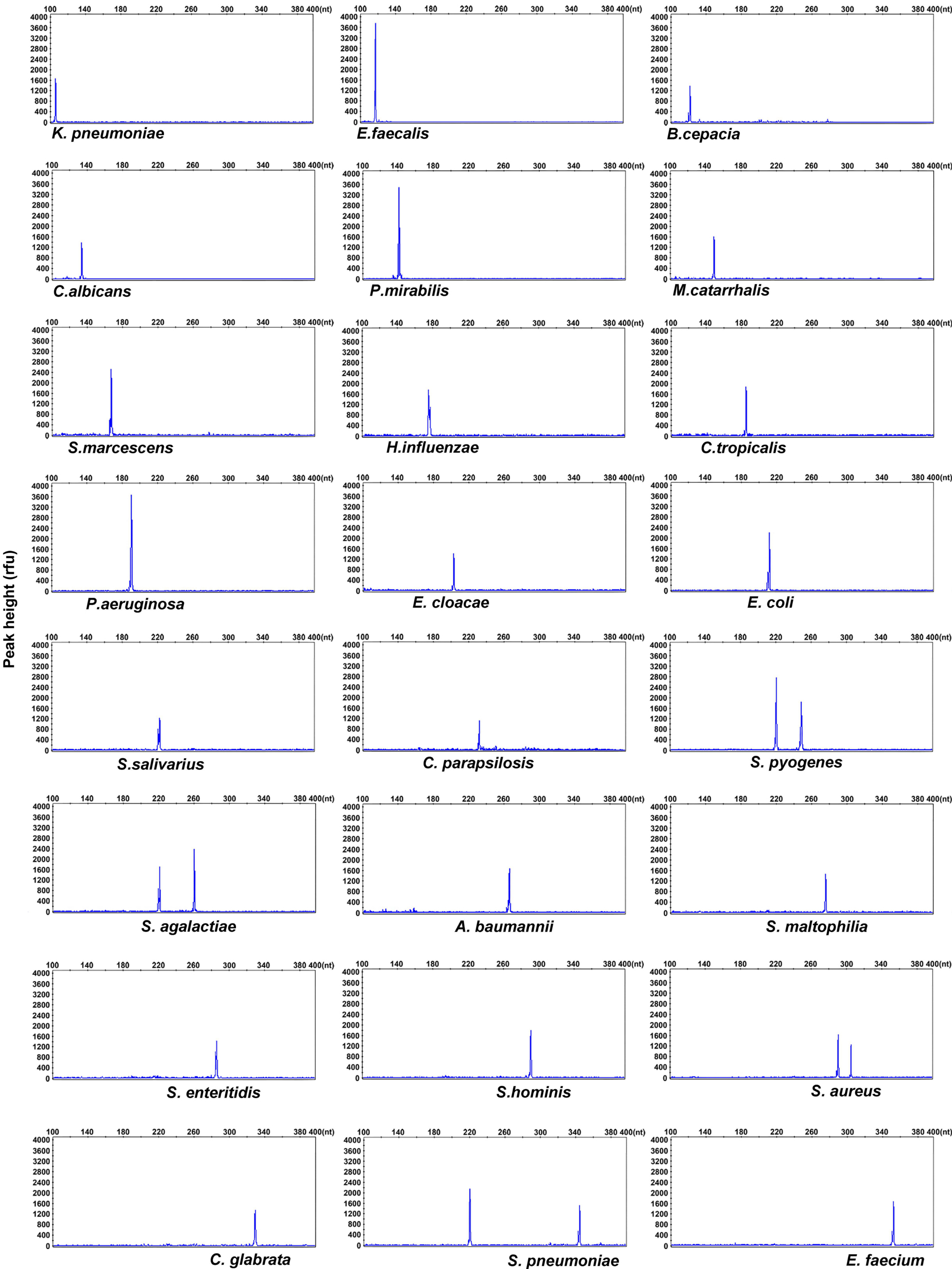
Peak height (rfu)
100 140 180 220 260 300 340 380 400(nt)
0 400 800 1200 1600 2000 2400 2800 3200 3600 4000
K. pneumoniae
E.faecalis
B.cepacia
C.albicans
P.mirabilis
M.catarrhalis
S.marcescens
H.influenzae
C.tropicalis
P.aeruginosa
E. cloacae
E. coli
S.salivarius
C. parapsilosis
S. pyogenes
S. agalactiae
A. baumannii
S. maltophilia
S. enteritidis
S.hominis
S. aureus
C. glabrata
S. pneumoniae
E. faecium

Figure S5. The BSI-HMGS has low LOD as verified using qualified bacterial/fungal suspensions

To determine the LODs of the BSI-HMGS in terms of quantified bacterial/fungal suspensions, 24 reference strains with tenfold serial dilutions, including 1.0×10^2–1.0×10^5 CFU/mL, were tested. All 24 targets could be detected at a concentration of 1.0×10^3 CFU/mL, showing a peak-height over the positive cut-off fluorescence signal of 500 rfu. Thus, the LOD of BSI-HMGS for bacterial/fungal suspensions was set at 1.0×10^3 CFU/mL. Abbreviation: LOD, limit of detection; HMGS, high-throughput multiplex genetic detection system.
